# A new dominant peroxiredoxin allele identified by whole-genome re-sequencing of random mutagenized yeast causes oxidant-resistance and premature aging

**DOI:** 10.18632/aging.100187

**Published:** 2010-08-13

**Authors:** Bernd Timmermann, Stefanie Jarolim, Hannes Rußmayer, Martin Kerick, Steve Michel, Antje Krüger, Katharina Bluemlein, Peter Laun, Johannes Grillari, Hans Lehrach, Michael Breitenbach, Markus Ralser

**Affiliations:** ^1^Next Generation Sequencing Group, Max Planck Institute for Molecular Genetics, 14195 Berlin, Germany; ^2^ Department of Cell Biology, University of Salzburg, 5020 Salzburg, Austria; ^3^ Department of Vertebrate Genomics, Max Planck Institute for Molecular Genetics, 14195 Berlin, Germany; ^4^ Institute of Applied Microbiology, University of Natural Resources and Applied Life Sciences, 1180 Vienna, Austria; * These authors contributed equally to this work

**Keywords:** Aging, whole genome resequencing, redox homeostasis, peroxiredoxin

## Abstract

The combination of functional genomics with next generation sequencing facilitates new experimental strategies for addressing complex biological phenomena. Here, we report the identification of a gain-of-function allele of peroxiredoxin (thioredoxin peroxidase, Tsa1p) via whole-genome re-sequencing of a dominantSaccharomyces cerevisiae mutant obtained by chemical mutagenesis. Yeast strain K6001, a screening system for lifespan phenotypes, was treated with ethyl methanesulfonate (EMS).  We isolated an oxidative stress-resistant mutant (B7) which transmitted this phenotype in a background-independent, monogenic and dominant way. By massive parallel pyrosequencing, we generated an 38.8 fold whole-genome coverage of the strains, which differed in 12,482 positions from the reference (S288c) genome. Via a subtraction strategy, we could narrow this number to 13 total and 4 missense nucleotide variations that were specific for the mutant. Via expression in wild type backgrounds, we show that one of these mutations, exchanging a residue in the peroxiredoxin Tsa1p, was responsible for the mutant phenotype causing background-independent dominant oxidative stress-resistance. These effects were not provoked by altered Tsa1p levels, nor could they be simulated by deletion, haploinsufficiency or over-expression of the wild-type allele. Furthermore, via both a mother-enrichment technique and a micromanipulation assay, we found a robust premature aging phenotype of this oxidant-resistant strain. Thus, TSA1-B7 encodes for a novel dominant form of peroxiredoxin, and establishes a new connection between oxidative stress and aging. In addition, this study shows that the re-sequencing of entire genomes is becoming a promising alternative for the identification of functional alleles in approaches of classic molecular genetics.

## Introduction

The free radical theory of aging implies that oxidative stress, and the generation of free radicals, are causally involved in the process of aging [1-3]. This theory is supported by many observations, including that yeast mother cells retain oxidatively damaged macro-molecules, whereas the daughter cells are formed from a juvenile set of proteins [4], or inherit functional enzymes whereas the damaged species are retained with the mother [5]. However, other discoveries challenge a causal implication of free radicals in different aging phenotypes [6]. Although most long-living mutants of *S. cerevisiae* and *C. elegans* tolerate high doses of oxidants, not all oxidant-resistant mutants are long living. As an example, both deletion of the metabolic regulator *AFO1* [7] and reduced activity of the metabolic enzyme *TPI1* [8] increase oxidant tolerances of yeast, but Δ*afo1* cells are massively long-living whereas TPI-deficient cells have a strong premature aging phenotype [7, 8]. In drosophila, mitochondrial ROS production correlates with aging but conversely, is not sufficient to alter lifespan [9]. All these observations are further complicated by the fact that mutants which are long-living under one environment/condition do not necessarily show this phenotype under other circumstances, i.e. yeast mutants with prolonged survival at 4°C are not enriched for mutants that are long-living at a higher temperatures [10]. In addition, there is a close relation of growth rate, metabolic activity and aging [11]. Since metabolism is itself a primary source for free radicals within the cell, it is difficult to distinguish between consequence and causality of oxidative damage during the aging process [6, 11]. Thus, although oxidative stress and free radicals are important players, their exact role during aging and the complex interplay of the involved genetic and biochemical components has yet to be clarified.

Systematic functional genetics/genomics is powerful in the identification of genetic components of biological processes and their interactions. With the introduction of systematic genetic libraries, such as an entire knock-out library of the yeast *S. cerevisiae* one decade ago [12], a new era of functional genetics began. Screening of systematic libraries allows circumventing the most time-consuming and limiting step of experimental genetics, which is the identification of the functional mutation.  Screens with the systematic libraries identified many components that influence yeast aging phenotypes [10, 13, 14]. However, all pre-made libraries have the disadvantage of being limited in the number of alleles they contain. In practical terms, genome-wide knock-out, over-expression and copy number variation libraries can be generated, but nothing such as genome-wide ‘point-mutation' libraries which would allow the isolation of alleles with new functionality. Therefore, although highly efficient, screens with systematic genetic libraries miss functional connections that can be identified by the isolation of new alleles in random-mutagenesis screenings. Thus, there is demand for experimental strategies that increase the efficiency of these classic approaches.

Here, we provide a case where whole-genome re-sequencing led to the identification of a new and dominant peroxiredoxin allele that causes oxidative stress resistance and premature aging. The W303-derived yeast strain K6001 [15], a model system that allows the enrichment of yeast mother cells [16], was used in a random mutagenesis experiment to isolate a mutant that exhibited strong resistance to the chemical oxidant cumene hydroperoxide (CHP). The mutant was dominant and segregated this phenotype in a regular way and independent of genetic background, observations which pointed to a monogenic trait. Using whole-genome re-sequencing of both strains by the Roche/454 system, and comparison of their genomes, we identified the mutation as a single nucleotide exchange in the gene, *TSA1*, coding for thioredoxin peroxidase (peroxiredoxin).

## Results

### Random mutagenesis and isolation of an oxidant-resistant K6001 mutant

Cell division of *S. cerevisiae* is asymmetric; mother and daughter cells are clearly recognizable by their size.  Mother cells can complete a limited number of cell cycles. At the end of this so-called *replicative* lifespan, the cells persist for some further time in a non-dividing state, and finally die, predominantly by apoptosis [17]. The yeast strain K6001 has been used to study this phenotype, because it facilitates the selective enrichment of yeast mother cells. On glucose media, daughters of K6001 do not express the essential *CDC6* gene, and are therefore unable to complete their cell cycle. Thus, the biomass which a K6001 culture achieves in glucose media, is a function of the number of daughters per mother and indicates its average replicative lifespan [16].

**Figure 1.**
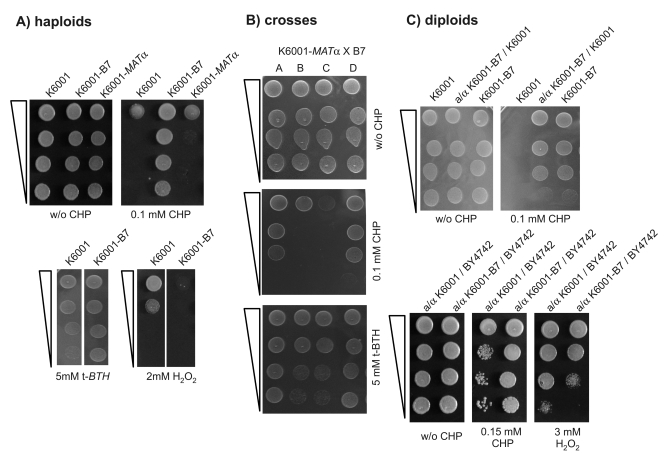
K6001-B7 carries a monogenic and dominant allele that confers resistance to cumene hydroperoxide (CHP). (**A**) *K6001-B7 is resistant to CHP.* Overnight cultures of the parent (K6001, *MAT*a), K6001-B7 and an isogenic *MAT*α (K6001-α32) strain were spotted as serial dilution on SC agar with and without CHP and grown at 28°C (upper panel). K6001-B7 grows on 0.1 mM CHP, a concentration lethal for K6001 and K6001-α32. (lower panel) Similar test were performed with oxidants tert-butyl hydroperoxide (t-*BTH)* (left) and H_2_O_2_ (right). (**B**) *K6001-B7 transmits with a monogenic Mendelian trait. *K6001-B7 was mated with K6001-α32, tetrads gained by sporulation and tested for CHP and t-BTH resistance. Shown is the 2:2 segregation of a representative tetrade. (**C**) *B7 is a dominant mutation. *(upper panel) K6001 and K6001-B7* were *mated with K6001-α32, resultant diploids assayed for CHP resistance. +/B7 diploids retained the stress resistance of the B7 haploid. (lower panel) *B7 is dominant across backgrounds.* K6001 and K6001-B7 were mated with the distant *S. cerevisiae* strain BY4742. Similar to the pure K6001 background situation, BY/K6001-B7 diploids were more resistant to CHP (left) and sensitive to H_2_O_2_ (right) compared to the BY/K6001 diploids.

To identify new genetic connections between aging and oxidative stress, we used K6001 in a random mutagenesis experiment. An exponential K6001 culture was mutagenized with ethyl  methansulfonate (EMS) and grown for two generations under non-selective conditions to enable mutation fixation. Mutagenized cells were then plated onto agar that contained the synthetic oxidant cumene hydroperoxide (CHP) at a concentration lethal for the wild-type strain. For every million of cells plated, an average of three colonies gained resistance and grew on the CHP-containing media. Obtained stress resistant mutants were tested for their replicative lifespan. We are presenting here one mutant (K6001-B7) that displayed resistance/sensitivity to multiple oxidants and clear regular 2:2 segregation in genetic crosses.  The isogenic mating partner, K6001α, was obtained by mating type switching of K6001 induced by a plasmid carrying the wild type HO gene. The mutant was mated to K6001α and the diploids were sporulated. Tetrads gained by sporulation were tested for resistance to CHP, t-butyl hydroperoxide (t-BHT), diamide, and hydrogen peroxide. Figure 1 summarizes the oxidant phenotypes of K6001-B7, the diploid obtained in the outcross just described, and the haploid progeny derived thereof (one typical tetrad). The mutant strain K6001-B7, the diploids obtained by outcrossing it to wild type, and the haploid progeny all showed the same phenotype: strong resistance to CHP, increased resistance to t-BHT, sensitivity to hydrogen peroxide (Figure 1A), and normal growth in the presence of diamide, the trait was inherited in a monogenic way (2:2 segregation in tetrads; Figure 1B) and the mutant allele was dominant even in crosses with a non-related wild type strain, BY4742 (Figure 1C lower panel).

### Identification of the K6001-B7 gene via whole-genome re-sequencing

Dominant mutations yielding stress-resistance are difficult to identify using classic yeast genetics; e.g. resistance phenotypes are commonly observed when anti-oxidant factors are over-expressed or over-active, leading to dominance of the mutant allele and limiting the possibilities and the specificity of complementation strategies with clone libraries that often over-express the inserted genes, even if they are centromeric. Furthermore, for yeast strains evolutionary distant from the reference genome of S288c, such as W303, the use of whole-genome-tiling arrays is limited due to potential unspecific hybridisation with primers designed for this distant genome.

Here, we decided on a whole-genome resequencing strategy using the Roche/454 platform to identify the B7 mutation. For this, we isolated genomic DNA from both K6001 and B7 and generated 454 sequencing libraries. Libraries were quality- controlled and sequenced by a Titanium sequencing kit (Roche). The run produced 662 MB of high-quality sequence at a median read-length of 527 bp (average read-length 499 bp). First, we aligned the sequence information to the S288c reference genome. 96.89 % of the coding and 96.83% of the noncoding regions were called at high quality in K6001 and 96,97%/96,98% in K6001-B7 (merged 97,36%/97,54%). Most of the non-called regions were neither sequenced in K6001 nor in K6001-B7, indicating that they were physically absent. (see Supplementary Figure 1 for an illustration of coverage uniformity). Based on this data, we calculated an average 19.2 fold coverage for K6001 and 19.5 fold coverage for K6001-B7. To compare the K6001/K6001-B7 genome with the S288c reference, we merged both sequence runs for an alignment, yielding an 38.8 fold total coverage. At this depth, we detected 12,482 SNVs and small (<50bp) insertions and deletions (that were sequenced at a minimum of 3x and on both strands) which distinguished K6001 from the reference genome. Surprisingly, although sequencing a haploid genome, we detected several mutations that had a calling rate between 5% and 95%. In a diploid genome, one would assume that these variants are heterozygotes. However, since we do not expect the existence of true heterozygotes in a haploid genome, we refer to these variants as presently unexplained non-uniform sequences. These variants were not entirely randomly distributed in the genome, many of these (62%) clustered close to the telomeric regions. This result could point to a natural variability of the genome in these regions. However, the result that the B7 phenotype was segregating in  2:2 manner  allowed  the  exclusion of these non-uniform SNVs from the candidate list. By setting a threshold of a minimal calling rate >95%, we reduced the list to 7213 uniform SNVs and small insertion/deletions.

**Figure 2.**
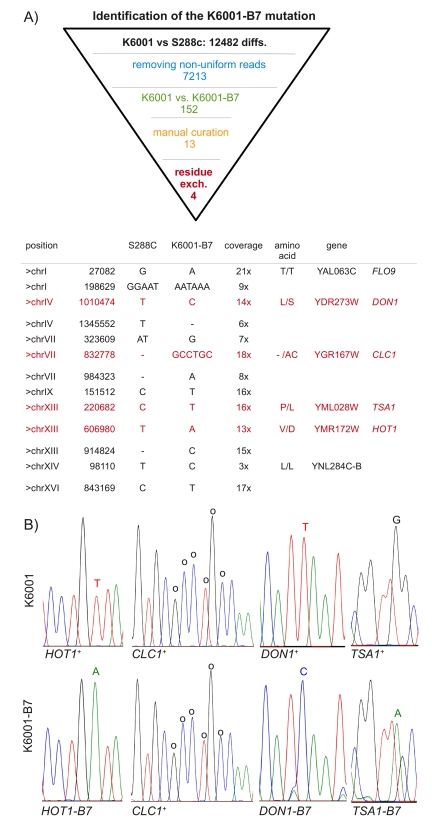
Identification of K6001-B7 by subtractive whole-genome resequencing. (**A**) 454 sequencing found 12,484 genetic differences between K6001 and the S288c reference genome. Four K6001-B7 candidates were identified by systematic narrowing of this list via the exclusion of non-uniform reads, substracting K6001 from K6001-B7, and the manual exclusion of alignment artefacts. Four of the remaining 13 variants were predicted to result in amino acid exchange. (**B**) Sanger resequencing of candidate regions. Mutant variants are highlighted. Please note that for *TSA1* the sequence trace of the reverse strand is shown.

We continued our investigations by comparing K6001 with B7 by removing all variants which were found in both genomes. This subtractive analysis narrowed our candidate list to 152 K6001-B7 specific mutations. Finally, we were able to remove alignment artefacts of the mapping algorithm by manual curation; primarily, some two-nucleotide transversions were wrongly called. In addition, we excluded all nucleotide variations which distinguished K6001-B7 from the reference genome, but were simply not sequenced at sufficient quality in K6001 and therefore potential false-positives.

The final list contained 13 candidate mutations. As illustrated in Figure 2, four of these were predicted to cause amino acid exchanges. We amplified these regions by PCR and subjected them to Sanger sequencing. The three SNV resulting in residue exchanges could be verified by capillary re-sequencing (Figure 2B). The fourth difference, a six nucleotide insertion of the *CLC1* gene, was also real, but found in both the K6001 and K6001-B7 strain.

### Oxidant resistance of B7 is mediated by a single residue exchange within the TSA1 gene

**Figure 3.**
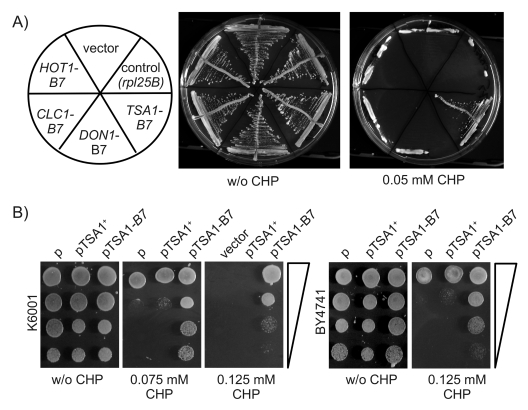
Ectopic expression of *TSA1-B7* mediates CHP resistance. (**A**) Candidate genes *HOT1, CLC1, DON1, *and* TSA1 *were amplified from the K6001-B7 genome, subcloned, and ectopically expressed in the K6001 parent. Transformants expressing *TSA1-B7* were viable on CHP media. (**B**) *CHP resistance is specific to the expression of TSA1-B7*. Centromeric plasmids encoding wild type *TSA1*^+^ and its B7 form were transformed into K6001. The additional copy of *TSA1^+^* had no effect on CHP resistance in K6001 (left) and S288c/BY4741 (right).

To identify the B7 gene among these candidates, we chose a classic strategy of cloning and phenotypic analysis. The four potential candidate genes were PCR-amplified from K6001-B7 genomic DNA, and sub-cloned into a yeast single-copy (centromeric) expression vector along with their endogenous promoter sequences. The plasmids subsequently verified by sequencing were transformed into K6001, and monoclonal descendants selected on SC Galmedia lacking histidine. As illustrated in Figure 3A, the transformants ectopically expressing *HOT1-B7*, *CLC1-B7*, *DON1-B7* as well those harbouring the control plasmids were viable on SC-His/Galmedia, but not more resistant  to CHP than the wild type.

**Table 1.** Oligonucleotide primers.

**CDS**	**fwd oligo**	**rev oligo**	**cloning**
*DON1*	TAGAATTC AGGGTACAGGCGAAGAAATG	TAGTCGAC CTACGTAAAACTTAATTCTT	*Eco*RI/*Sal*I
*CLC1*	GAAGAGCT CAACAATACAATAAACCCAATC	TGGTCGAC TTAAGCACCGGGAGCCTTCG	*Sac*I/*Sal*I
*TSA1*	GAGAGCTC ATACGCTACCCAAGTACAGAAG	TGTCTCGAG TTATTTGTTGGCAGCTTCGA	*Sac*I/*Xho*I
*Hot1*	GAGAGCTC ATTATATCCATGTTAAGTTCG	TATCTCGAG CTATATTCCAGCAAGGCTCT	*Sac*I/*Xho*I
	Underlined sequences represent introduced restriction sites		

**Table 2.** MRM transitions.

	Q1/Q3 transition	sequence	Charge/Fragment ion
TSA-pep_1	617.85 - 984.55	HITINDLPVGR	2+ / y9
TSA-pep_2	451.77 - 732.43	GLFIIDPK	2+ / y6
TPI-pep_1	762.37 -989.49	ASGAFTGENSVDQIK	2+ / y9
TPI-pep_2	758.93 - 864.46	KPQVTVGAQNAYLK	2+ / y8

In contrast, the transformants of the *TSA1-B7 *encoding plasmid grew perfectly well on media containing 0.05 mM CHP. To exclude in a second step, that this phenotype was the result of a gene dose effect caused by the extra copy of *TSA1*, we generated an additional, isogenic plasmid encoding for its wild type form as well. Along with the empty as well as the *TSA1-B7* vector, this plasmid was transformed into K6001 and the S288c derived BY4741 strain. Selected trans-formants were grown over night, and spotted as dilution series on agar plates with and without the oxidant. As illustrated in Figure 3B, only yeast containing the *TSA1-B7* plasmid, but not its wild-type form, were resistant to CHP. This phenotype was also observed in the S288c (BY4741) background, confirming the dominant and background-independent inheritance. Thus, a new dominant allele in *B7,* encoding for Tsa1p^Pro182Leu^, was responsible for the increased oxidant tolerance of the K6001-B7 mutant.

**Figure 4.**
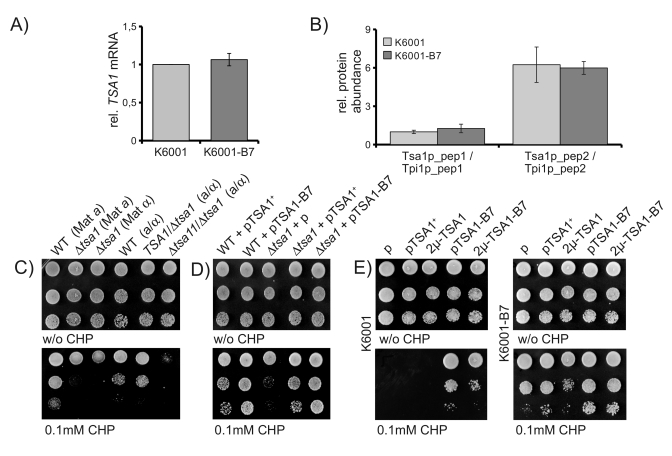
CHP resistance is mediated by a *TSA1* gain of function. (**A**) *TSA1 mRNA expression does not differ between K6001 and K6001-B7. TSA1 *mRNA levels were assayed by classic qRT-PCR and normalized to the geomean of reference transcripts *ACN9, ATG27 *and* TAF10*. (**B**) *Tsa1p protein levels do not differ between K6001 and K6001-B7. *Two Tsa1p specific tryptic peptides were quantified by nanoLC-MS/MS and set in relation to two peptides of triosephosphate isomerase (Tpi1p) in both K6001 and K6001-B7 extracts. (**C**) *Deletion of tsa1, but not TSA1/Δtsa1heterozygosity, decreases CHP tolerance. Tsa1* deletion strains of* MAT*a and *MAT*α mating types, as well as corresponding heterozygote and homozygote diploids of the S288c background, were spotted on CHP containing agar and grown at 28°C. (**D**)* CHP sensitivity of Δtsa1 is restored upon ectopic expression of TSA1^+^. MAT*a *Δtsa1 *cells were transformed with single-copy expression vectors encoding *TSA1+* and *TSA1-B7* and assayed for growth on CHP containing media. (**E**) *Mulicopy overexpression of TSA1^+^ does not mimic TSA1-B7, heterogeneous overexpression of TSA1^+^/B7diminishes the oxidant resistance phenotype. *K6001 (left) and K6001-B7 (right) were transformed with single-copy (p) or high-copy (2μ) *TSA1*^+^ and *TSA1-B7* expression plasmids, spotted on CHP containing yeast agar, and  grown at 28°C for three days.

To gain insights, if *TSA-B7* represents a gain or a loss of function allele, we first assayed *TSA1-B7* mRNA levels in the K6001 parent and its B7 mutant. As shown in Figure 4A, via quantitative RT-PCR, we could not detect any difference in *TSA1* mRNA expression between the K6001 parent and K6001-B7. Next, using targeted mass spectrometry, we addressed Tsa1p protein levels. By quantifying two Tsa1p-specific tryptic peptides (GLFIIDPK  and HITINDLPVGR) and nor-malization of their peak areas to peptides specific for Triosephosphate isomerase (ASGAFTGENSVDQIK and KPQVTVGAQNAYLK), we found that Tsa1p is expressed at identical levels in K6001 and K6001-B7 (Figure 4B). Thus, stress resistance caused by *TSA1*-B7 is not attributable to altered expression of Tsa1p.

Next, we questioned, if the stress resistance of B7 might be attributable to loss of function of Tsa1p. As shown in Figure 4C left, deletion of the *TSA1* gene in two haploid yeast strains decreased rather then increased yeast's resistance to CHP. Thus, depletion of *TSA1* does not result in the *TSA-B7* phenotype, indicating that the dominant*TSA1-B7* is not a loss of function allele. Next, we spotted diploid wild-type, *TSA1*/*Δtsa1* heterozygous and *Δtsa1*/*Δtsa1* homozygous strains on oxidant-containing agar. Whereas the homozygous *Δtsa1* / *Δtsa1* deletion strain was sensitive to CHP, this phenotype was not detected in the *TSA1*/*Δtsa1 *heterozygotes (Figure 4C, right). Thus, partial loss of *TSA1 *due to a haploinsufficiency does not resemble the B7 phenotype.

We continued by addressing the effects of ectopic Tsa1 and Tsa1-B7 expression in the *Δtsa1* background. BY4741 based *Δtsa1 *yeast was transformed with *TSA1-* and *TSA1-B7*- as well as empty single-copy (CEN) plasmids, and assayed for CHP resistance. Expression of *TSA1* in the deficient background restored the wild-type phenotype, whereas B7 expression resulted in CHP resistant transformants (Figure 4D).

Finally, we questioned if the increased CHP resistance might be attributable to higher Tsa1 activity and thus, could be simulated by overexpression of wild-type Tsa1p. We subcloned *TSA1* and *TSA-B7* into a high-copy 2μ plasmid (p423GPD) and transformed both K6001 and K6001-B7. As shown in Figure 4E, overexpression of wild-type *TSA1* did not simulate the effects of the B7 mutation. Unexpectedly, however, wild-type K6001 cells harbouring the multicopy overexpression plasmid for *TSA1-B7* resulted in less CHP resistant cells than single-copy counterparts (Figure 4E left). This effect was not seen in the mutant background (Figure 4E right). However, here similar effects were caused by multicopy expression of the wild-type allele. Thus, heterogeneous overexpression of *TSA1* with *TSA1-B7* reduces the stress resistance mediated by the *TSA1-B7 *allele*.*

In sum, these results indicate that TSA1-B7 is a gain of function allele. *TSA1-B7* is dominant above *TSA1* across yeast backgrounds, its phenotype is not simulated by *TSA1* over- or underexpression nor its deletion, and finally, heterogeneous *TSA1/TSA1-B7* overepxression diminishes the stress resistant phenotype.

### K6001-B7 has a premature aging phenotype

**Figure 5.**
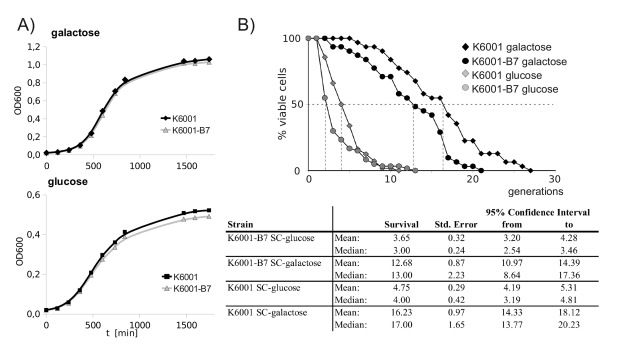
K6001-B7 has a shortened replicative lifespan. (**A**) K6001 and K6001-B7 have similar growth in galactose (upper panel), but K6001 stagnates at a lower biomass in glucose media (lower panel). (**B**) Lifespan assay by micromanipulation. Daughter cells from K6001 and K6001-B7 were continuously removed by micormanipulation and counted (upper panel) and analyzed statistically (lower panel). The shortened lifespan of K6001-B7 in both glucose and galactose media was tested statistically significant using Mantel-Cox, Breslow as well as Tarone/Ware statistics.

Since altered resistance to oxidants has often been observed in yeast strains with aging phenotypes, we screened for potential alterations in the replicative lifespan of the B7 mutant. K6001 offered the possibility to screen for alterations replicative aging via the generation of growth curves in galactose- and glucose, an alternative to time-consuming micromanipulation experiments [16]. As illustrated in Figure 5A, K6001 and K6001-B7 both showed a comparable doubling time in galactose. However, when shifted to glucose, linear growth stagnated at a lower biomass in K6001-B7. The final biomass reached by the wild type and mutant was significantly different. This indicated that - although oxidant-resistant - K6001-B7 has a premature aging phenotype. To confirm this result, we assayed replicative aging of K6001 via removal and counting of daughter cells by micromanipulation (Figure 5B). On galactose media, the median lifespan of K6001 was 17 generations, and the lifespan of K6001-B7 shortened to 13 generations.

The genetic manipulations of K6001 dramatically shorten its lifespan on glucose media [16]. Nonetheless, we also compared the lifespan of K6001 and K6001-B7 mothers under these conditions. Here, the median replicative lifespan was 3 for K6001-B7, and 4 generations for K6001. Thus, the premature aging phenotype of K6001-B7 is not galactose-specific. Differences in the aging phenotypes on both media were statistically significant as tested by Log Rank-, Breslow- and Tarone Ware statistics.

## Discussion

Here, we describe the generation, identification and initial functional analysis of a dominant peroxiredoxin allele which causes oxidative stress resistance and premature aging in yeast. The oxidant-resistant mutant was isolated after EMS treatment of the yeast aging model strain K6001, a descendant of the broadly used yeast strain W303. Subsequent experiments demonstrat-ed that B7 is transmitted in a monogenic and dominant pattern.

The classical genetic and molecular genetic approach for the dissection of a dominant monogenic trait is constructing a genomic clone bank representative for the genome of the mutant, transforming this bank into wild type cells, selecting for clones that show the phenotype (in our case, CHP resistance), cloning and subcloning, and identifying the gene by capillary sequencing (see [18] for an overview on classic screenings methods). For the case presented here, this strategy would be burdened by several limitations, like occasionally a non-selectable phenotype, or if transformats from the clone bank confer a selectable phenotype even if they contain overexpressed wild type genes. A second classical strategy is cloning via mapping using determination of linkage with a large number of genetic markers on all chromosomes in meiotic tetrads. This strategy, however, is tremendously laborious. Here, we tested if with the advent of ever improving sequencing methods, resequencing of whole yeast genomes has become a serious alternative method to isolate and confirm the gene in which a given dominant mutation resides.

The genome of both, the parent strain and the mutant derived from it, were sequenced using the Roche/454 system. At an average read length of 499bp, this yielded a 38.8 fold average total coverage of the genome, and divided into 19.2X coverage for K6001 and 19.5X coverage for K6001-B7.Compared to the S288c reference, we detected 7660 uniform small nucleotide variations. 7499 of these, proven by the very stringent criteria of a >10X coverage are available in the Supplementary material. This rate indicates one nucleotide exchange per 1600 bp, which points to a high degree of evolutionary divergence between S288c and W303/K6001. Interestingly, the rate of divergence differed between the individual chromosomes (Supplementary Figure 2).

An interesting observation of this study was the identification of many non-uniform variants in the haploid genome. Many of these were called with high coverage and confidence, indicating that they might be biologically relevant and not the result of technical artefacts. 62.1 % of the variants detected at a calling rate between 25 and 75% were located in or close to the telomeric regions; however, they clearly were located in unique sequences. We will intensively discuss the nature of these variants in a future publication.

In the last three years, next generation sequencing has been used for mapping of epigenetic mutations [19], identification of spontaneous mutations [20], or for evolutionary considerations [21] in yeast. The accuracy of next generation technologies is key to this approach and in the course of this study we identified less than 1 difference (0.86) in 1,000,000 nucleotides between both genome sequences. Lynch et al. [20] used a pyrosequencing approach and reported an average depth of sequence coverage of 5X and restricted their analyses to sites within each genome with at least 3X coverage. The authors pointed out that especially for homopolymeric sites, a higher coverage depth is necessary. Recently, Araya et al. [22] used a short read sequencing-by-synthesis approach for their whole genome sequencing of a laboratory-evolved yeast strain. At an average depth of 28X, they covered 93% of the yeast genome. The coverage required for an in-depth analysis of individual genomes highly depends on the used sequencing technology. According to Wheeler et al. [23] sequencing of diploid organisms demonstrated a minimum of 15X coverage for pyrosequencing, and a 30X for sequencing-by-synthesis for accurate detection of heterozygous variants. For haploid genomes, the minimal coverage depth required is lower. In this study we reached a calling rate of 98% of the genome with an average coverage of 19.4X for both strains. For the detection of single nucleotide variations which distinguish K6001 from the reference genome, we merged the genomes of both K6001 and K6001-B7, which resulted in a 38.8x fold average genome coverage.

The number of detected variants which distinguished our strain from the Reference genome (12,482) was substantially large. To delimit candidate genes to identify the mutation causing the phenotype observed, we used a rigorous subtraction strategy. Surprisingly, the 12,482 SNV contained 5269 non-uniform variations which had a calling rate < 95 %. We excluded these non-uniform SNVs, because such a frequency would be incompatible with the observed 2:2 segregation of the B7 mutant. Next, we removed all variations that were shared between K6001 and K6001-B7, and all differences to the S288c genome that were not present in the K6001 genome sequence. At the end, we curated the final list manually to identify alignment artefacts of the mapping algorithm. We ended with a list of 13 differences, of which 6 were located in protein coding regions. Four of these mutations were predicted to cause residue exchanges.

Therefore, EMS mutagenesis had caused one mutation per ~950,000 nucleotides in the genome, and thus, indicated that the applied protocol was efficient to create a monogenic trait. However, we have to consider that mutant B7 was isolated after strong selection on CHP, and therefore the unbiased primary mutation rate immediately after EMS mutagenesis could have been substantially different.

We started the experimental verification strategy focusing on the four residue exchanging mutants. This choice was hypothesis driven; at this stage of the project, also non-exonic sequences were potential, although less probable, candidates for the B7 gene. We amplified *CLC1*, *DON1*, *HOT1* and *TSA1* genes by PCR from both K6001 and K6001-B7. Sanger sequencing verified the B7-specific mutations in *DON1*, *HOT1*, and *TSA1;* the six nucleotide insertion (relative to S288c) into *CLC1* was also real, but found in the parent strain as well. These four genes were then subcloned with their native promoters into a single-copy yeast expression vector, transformed into strain K6001, and transformants tested for their phenotype on CHP containing media. These experiments identified and confirmed *TSA1-B7*as the phenotype*-*causing gene.

Tsa1p (*t hiol s pecific a ntioxidant*), a 2-Cys peroxiredoxin, is an important enzyme of the cellular antioxidative machinery and catalyzes H_2_O_2_ reduction in the presence of thioredoxin, thioredoxin reductase and NADPH [24]. Tsa1p furthermore acts as a molecular chaperone, and is associated with ribosomes, it prevents oxidative damage of newly synthesized polypeptides [25]. It was previously shown that disruption of Tsa1p diminishes oxidative stress resistance of yeast; cells lacking this enzyme were highly sensitive to tert-butyl hydroperoxide, hydrogen peroxide, and CHP [26]. Peroxiredoxin 1 has been associated to the aging of mammals, since it interacts and stimulates the activity of the lifespan regulator protein p66Shc [27], and disruption of *Tsa1p *in yeast shortens its chronological lifespan [28]. The results presented here show that the involvement of Tsa1 in aging phenotype is complex. In contrast to the *TSA1* knock-out, yeast cells expressing the *TSA1-B7* allele gain resistance to oxidants, but are also compromised in a lifespan phenotype. Thus, changes in the natural antioxidative capacities of the peroxiredoxin system in either direction can accelerate yeast aging. These results add and substantiate other observations that highlight the importance of a natural redox balance, rather than the total antioxidative capacity, as an important determinant of cellular lifespan [29-31]. For instance, *C. elegans *requires a natural rate of free radical generation for lifespan extension by caloric restriction [32], and yeast cells, which are oxidant-resistant due to an excessive NADPH production caused by mutations in triose phosphate isomerase gene, are also replicatively short lived [8]. Thus, understanding the influence of free radicals and oxidative stress on the complex phenotype of aging requires examination of these processes in the context of the highly evolved regulation of the cellular redox environment.

Our study demonstrates that whole-genome re-sequencing is suitable to identify a functional single nucleotide exchange generated by random mutagenesis. Although all commercially available sequencing platforms (Genome Analyzer (Illlumina), SOLiD (Life Technologies) and FLX Genome Sequencer (454/Roche)) would provide appropriate workflows, we decided on 454 sequencing because of large read-lengths, sequence accuracy and the available software tools. All data was collected in a single Titanium run on the FLX sequencer. Thus, whole genome re-sequencing strategies have the potential to increase the efficiency and flexibility of random strategies, highly increasing their attractiveness for addressing current biological problems.

## Materials and methods

Yeast cultivation and mutagenesis. Yeast was cultivated on yeastextract peptone dextrose (YPD) or galactose (YPGal), synthetic complete glucose (SC) or galactose (SCGal) media at 28°C. For EMS mutagenesis, logarithmically growing K6001 cells were washed twice with 50 mM potassium phosphate (pH 6.8), and resuspended in 10 ml of this buffer supplemented with 300μl EMS. After one hour incubation at 28°C, where 10% of the cells were still alive, mutagenesis was stopped by adding 10ml 10% w/v sodium thiosulfate [33].

qRT-PCR. Yeast total RNA was extracted using RiboPure-Yeast Kit (Ambion). After quality control, cDNA was synthezised using 12-18 oligo dT primers and Moloney Murine Leukemia virus (*M-MuLV*) reverse transcriptase (NEB) according to the manufacturer's instructions. Real-time PCRs were performed in triplicates in a final volume of 5 μl containing 1 μl cDNA, 1 μl 5x combinatorial enhancer solution (CES) [34], 0.5 μl primer mix and 2.5 μl 2× SybrGreen master mix (Fermentas). Reactions were run on a Prism 7900HT sequence detection system (ABI). The thermal cycling conditions comprised 50°C for 2 min, 95°C for 10 min, and 40 cycles of 95°C for 15 s/60°C for 1 min. The relative expression ratio of the target gene *TSA1* was normalized to the geometric mean of three endogenous reference transcripts (*ACN9*, *ATG27, TAF10*) by the method of Pfaffl [35].

Cloning and sanger sequencing. Candidate genes were amplified by oligonucleotides given in table 1 using Phusion polymerase (Finnzymes). The resulting products were gel-purified and used a) for Sanger resequencing and b) subcloned into a pRS413-derived yeast single-copy centromeric expression vector. For this, the products were treated with the endonucleases (New England Biolabs) given in Table 1, and ligated into compatible sites of the yeast vector. 2μ overexpression plasmids p423-*TSA1* and p423-*TSA1-B7* were generated by excision of the corresponding fragments with *Sac*I/*Xho*I from the cen-plasmid, and their ligation into the backbone of the *Sac*I/*Xho*I digested 2μ vector p423GPD [36]. Plasmids were verified by endonuclease digestion and sequencing.

Targeted protein quantification by mass spectrometry. Tsa1p levels were quantified by the means of determination of their relative abundance relative to the expression of triosephosphate isomerase (Tpi1p). Whole-proteome tryptic digestes were generated and analyzed by nanoflow liquid chromatography tandem mass spectrometry (nanoLC-MS/MS) on an QTRAP5500 hybrid triple quadrupole/ion trap mass spectrometer (AB/Sciex) as described earlier [37]. Analyzed peptides and the MRM transitions used for quantification are given in Table 2.

Lifespan assay by micromanipulation. Logarithmically growing yeast cultures were plated at low density, and at least 50 daughter cells were isolated as buds with a MSM micromanipulator (Singer instruments). After their first division, mothers were removed and 2^nd^ generation virgin cells kept for analysis. The lifespan of these cells was determined by counting and removing all subsequent daughters at 28°C. Cells were shifted to 8°C overnight to allow resting of the investigator; depending on the age of the cells, 1-2 generations were completed at this temperature per night. Statistical calculations for lifespans were conducted with SPSS 11.0 (SPSS) and Excel with Winstat. By applying Kaplan-Meier statistics the standard deviations of the median lifespan at a confidence level of 95% were calculated. To show if two given survival distributions are significantly different at a 95% confidence level, logrank (Mantel-Cox), the modified Wilcoxon test statistic (Breslow), and Tarone & Ware statistics were used as described earlier [16].

454 Sequencing and data analysis. DNA was sheared by sonication to a fragment size of 500 - 800bp, and adaptors ligated. The amplified template beads were recovered after emulsion breaking and selective enrichment. Sequencing primer was annealed to the template and the beads were incubated with *Bst* DNA polymerase, apyrase, and single-stranded binding protein. Template beads, enzyme beads (required for signal transduction) and packing beads (for *Bst* DNA polymerase retention) were loaded into the wells of a 70 x 75 mm two compartment picotiter plate. The picotiter plate was inserted in the flow cell and subjected to pyrosequencing on the Genome Sequencer FLX instrument (454/Roche).

The system flows 200 cycles of four solutions containing either dTTP, alphaSdATP, dCTP or dGTP reagents, in that order, over the cell. For each dNTP flow, a single image was captured by a charge-coupled device (CCD) camera on the sequencer. The images were processed to identify DNA bead-containing wells and to compute associated signal intensities. The images were further processed for chemical and optical cross-talk, phase errors, and read quality before base calling was performed for each template bead.

Raw data processing. After default raw data processing, we used a resequencing trimming filter to increase the data output. (parameters doValleyFilterTrimBack = false, vfBadFlowThreshold = 6, vfLastFlowToTest = 168, errorQscoreWindowTrim = 0.01).

Mapping 454 reads to a reference genome. We generated 1.3 million sequences which produced 662 million bases that were aligned to the *Saccharomyces cerevisiae* reference genome [38], using GS reference mapper version 2.3. The best match in the genome was used as the location for the reads with multiple matches. SNPs and small insertion-deletions were included in the analysis.

Filtering small nucleotide variations. To identify the phenotype-causing SNP of the B7, we first removed non-uniform SNVs and insertions/deletions by introducing a cutoff-filter of <95%.  Next, we eliminate those variants which were equal in B7 and K6001. The remaining 152 differences were controlled by hand, taking into account the covered position of the yeast genome, the reference base and the consensus base for each position (‘454AlignmentInfo' file). Of these, we manually compared all variants called with at least three non-duplicate reads in both directions with the consensus base position in K6001.

## Supplementary data

Supplementary Figure 1

Supplementary Figure 2

Supplementary Table 1
