# Supplementary material for: A new dominant peroxiredoxin allele identified by whole-genome re-sequencing of random mutagenized yeast causes oxidant-resistance and premature aging
Source: Aging (Albany NY). 2010 Aug 13;2(8):475–86. doi: 10.18632/aging.100187 (PMC2954039; doi:10.18632/aging.100187)
Supplement: Supplementary Figure 1 [file aging-02-475-s001.pdf]

# Supplementary figure 1: coverage uniformity

The 16 yeast chromosomes were split into virtual 1kb units, and we calculated the average coverage for each of these units. These numbers are shown aligned to the Reference genome.

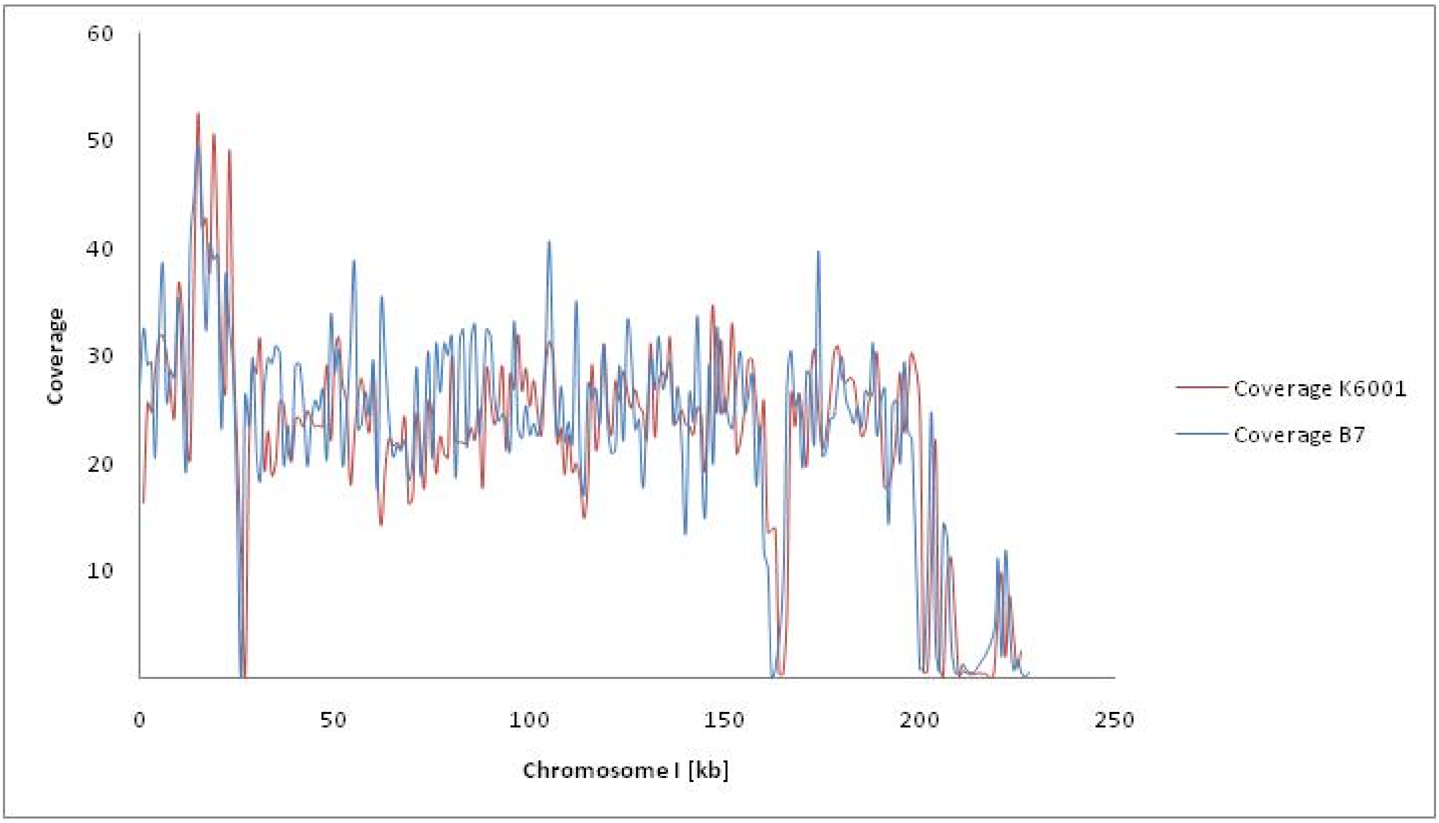


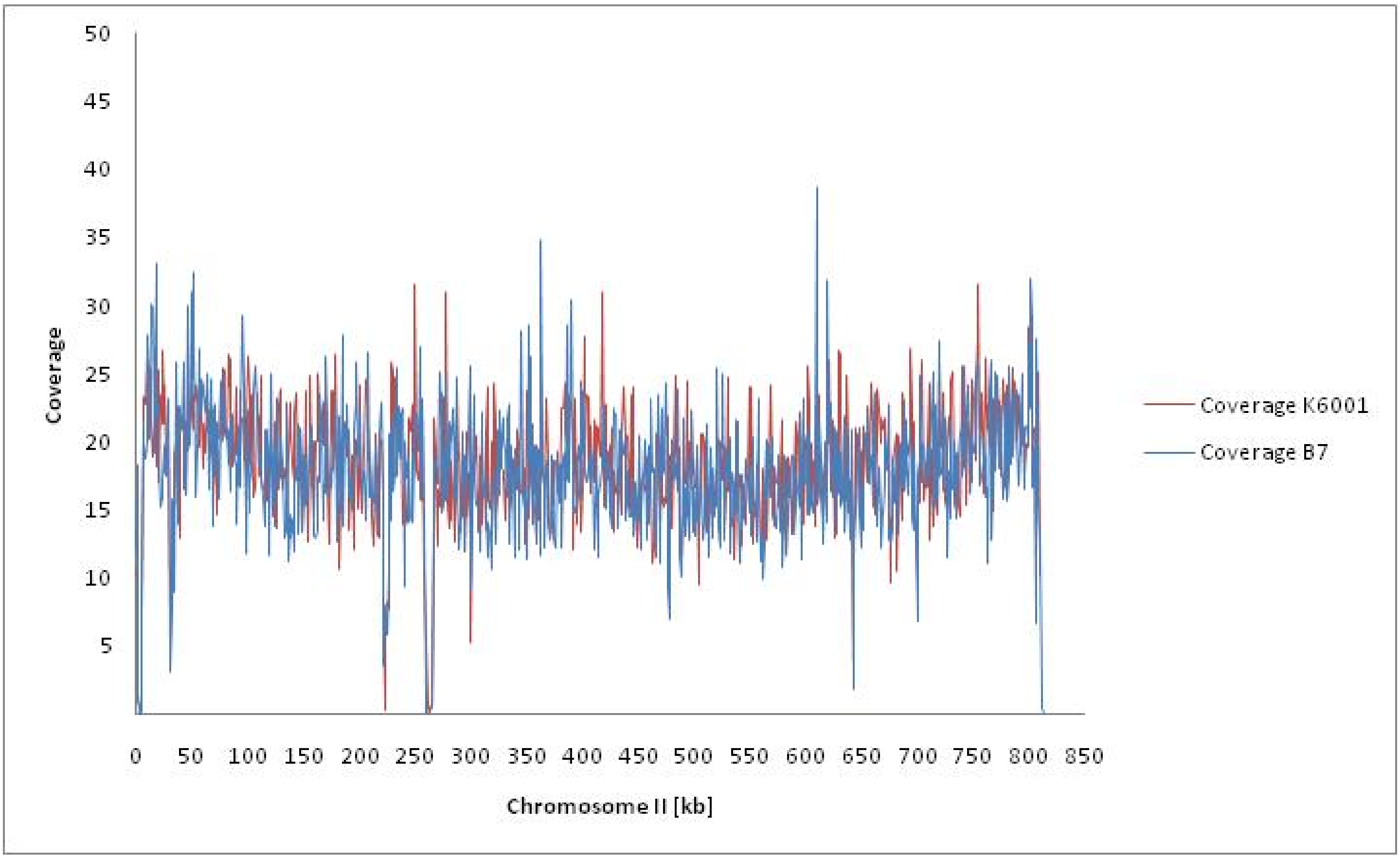

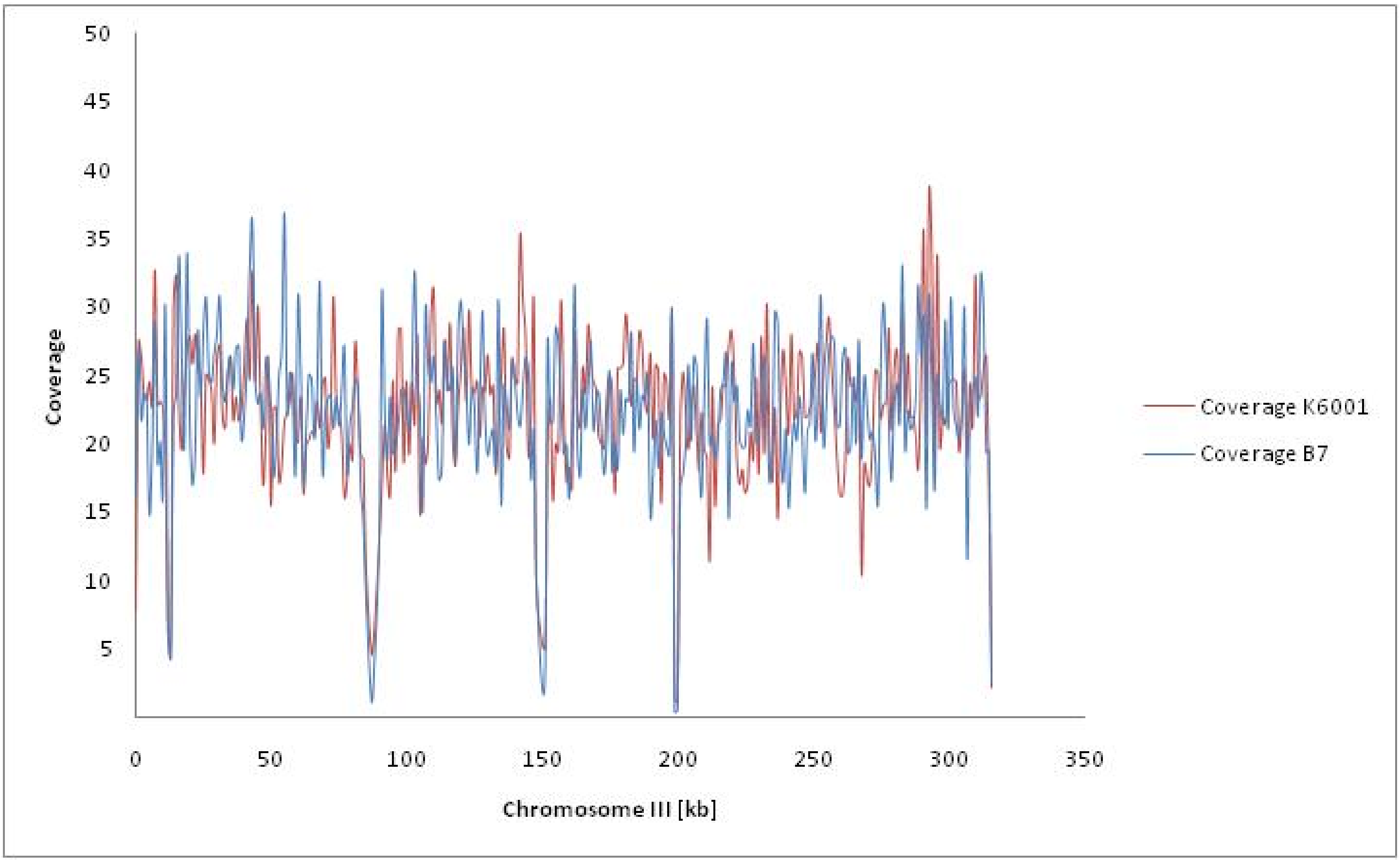

Coverage
Coverage K6001
Coverage B7
Chromosome III [kb]


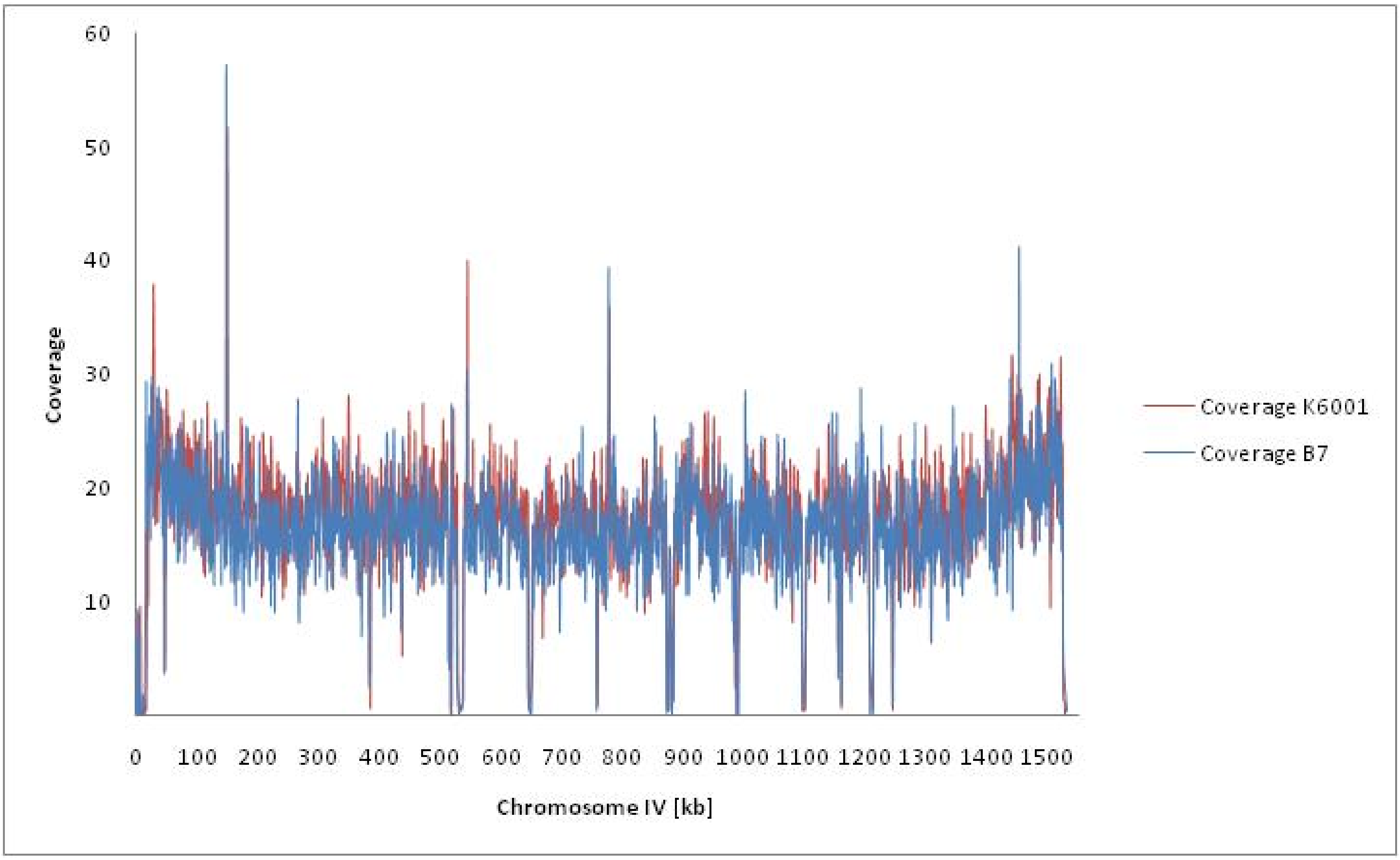

Coverage
Coverage K6001
Coverage B7
Chromosome IV [kb]

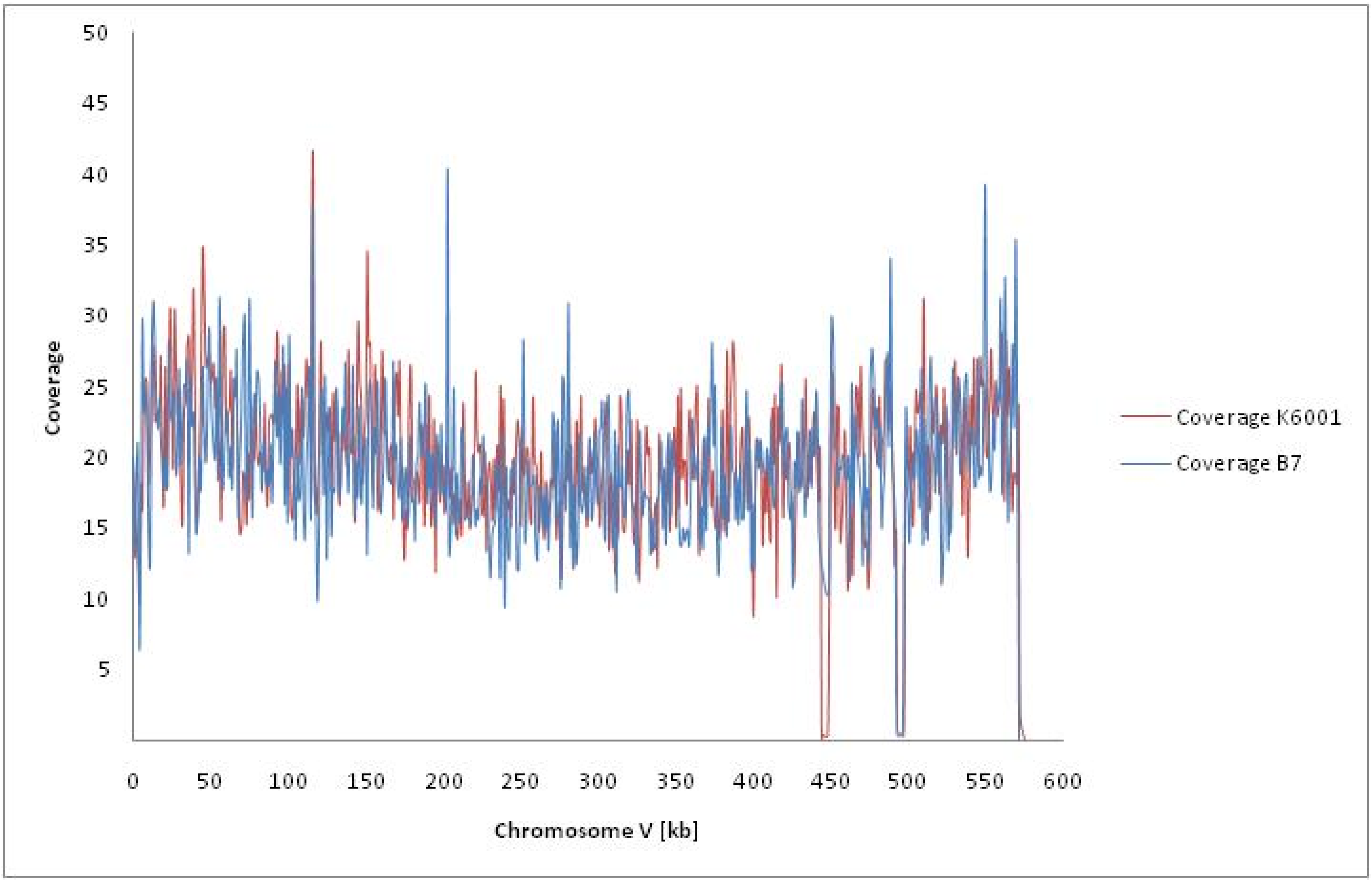

50
45
40
35
30
25
20
15
10
5
Coverage
0 50 100 150 200 250 300 350 400 450 500 550 600
Chromosome V [kb]
Coverage K6001
Coverage B7


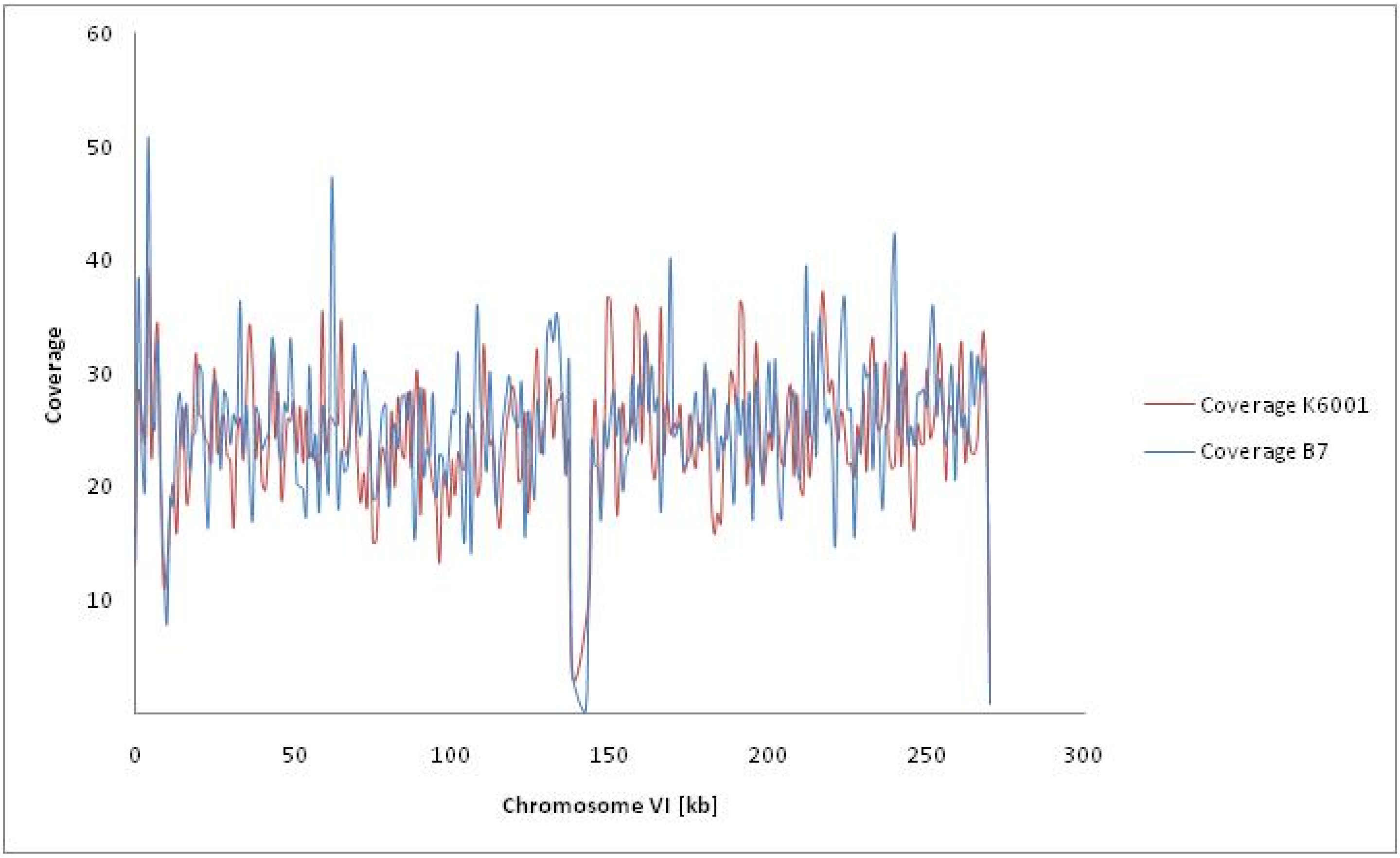

60
50
40
30
20
10
Coverage
0 50 100 150 200 250 300
Chromosome VI [kb]
Coverage K6001
Coverage B7

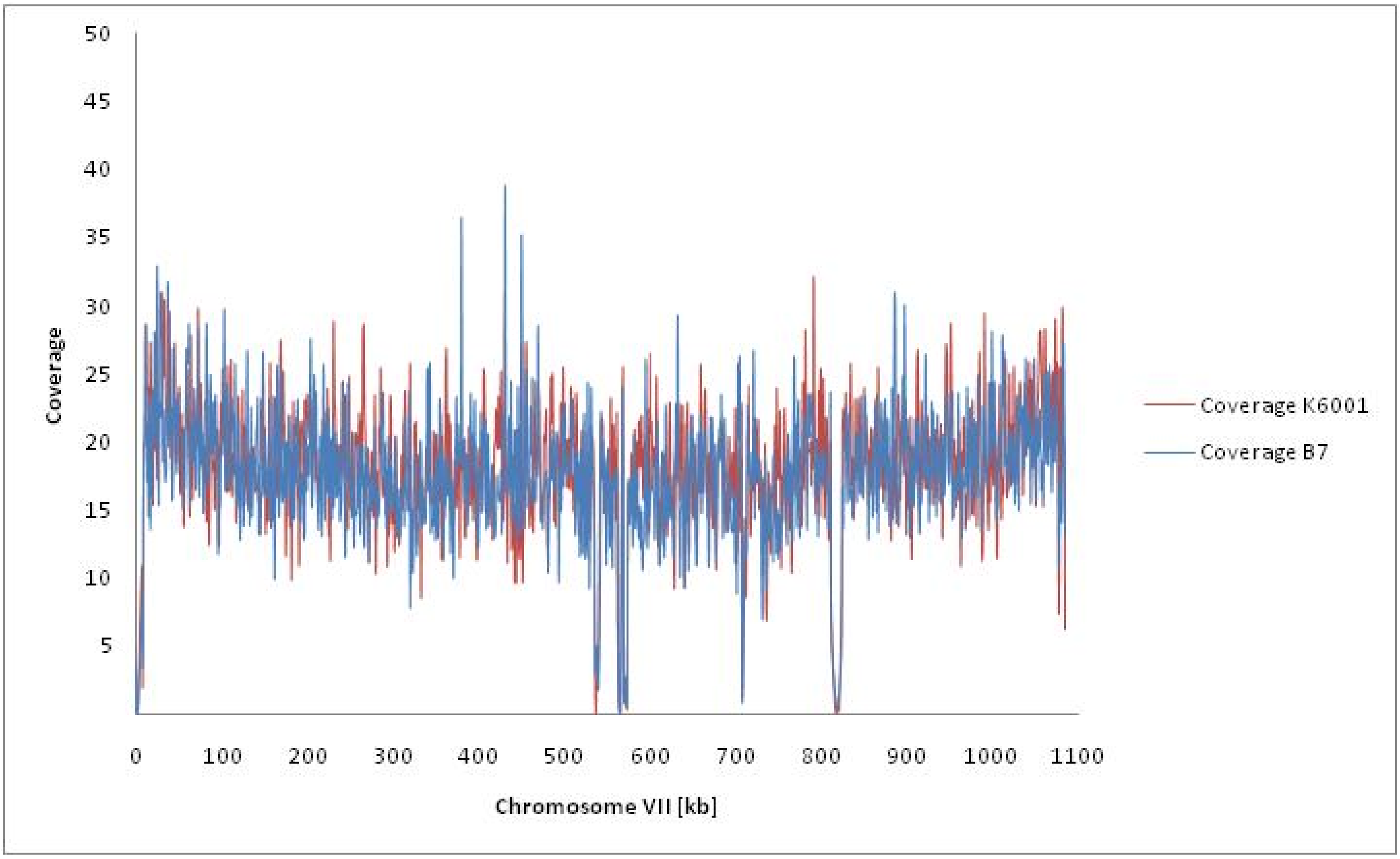
Coverage
Chromosome VII [kb]
Coverage K6001
Coverage B7

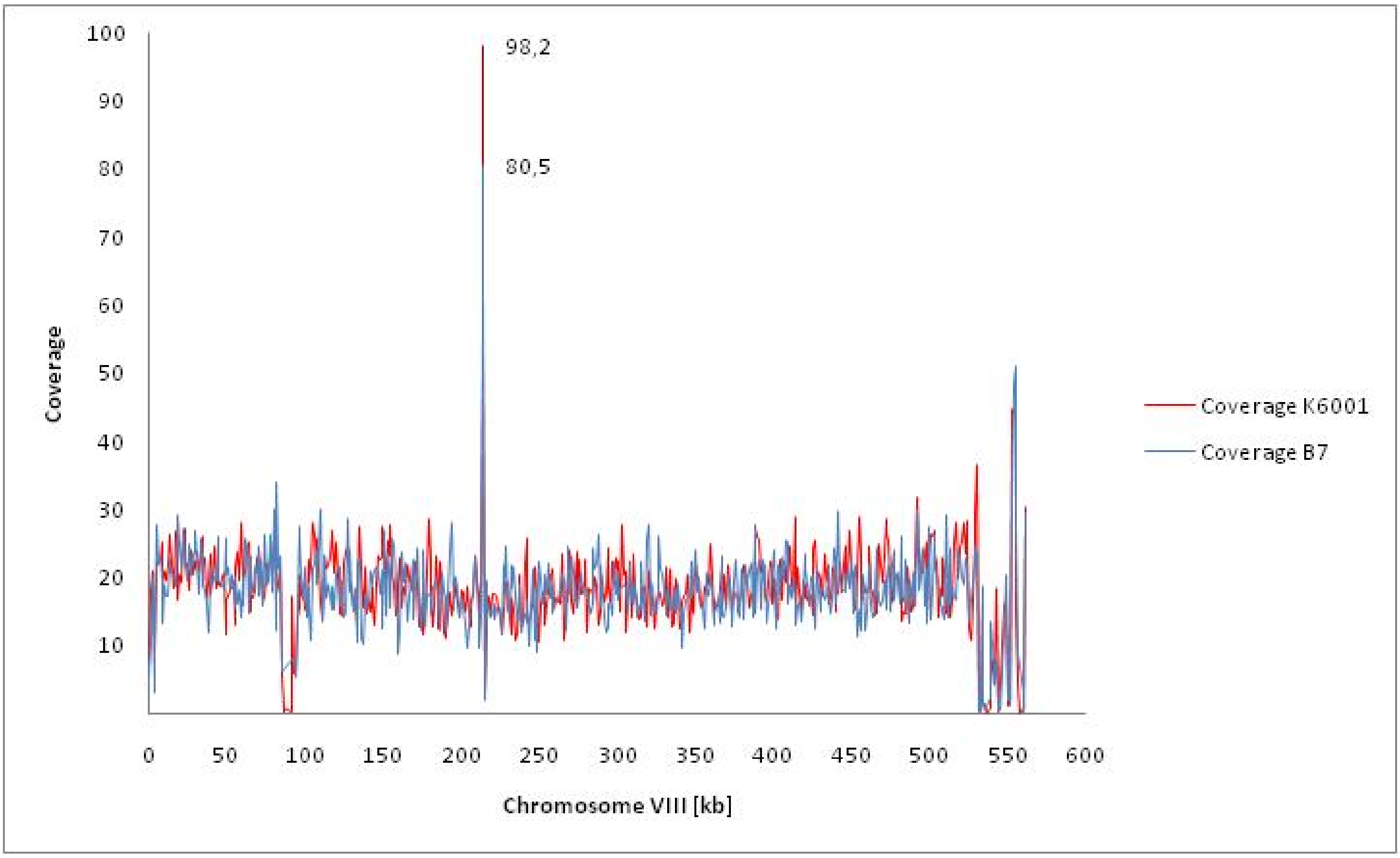
98,2
80,5
Coverage
Chromosome VIII [kb]
Coverage K6001
Coverage B7

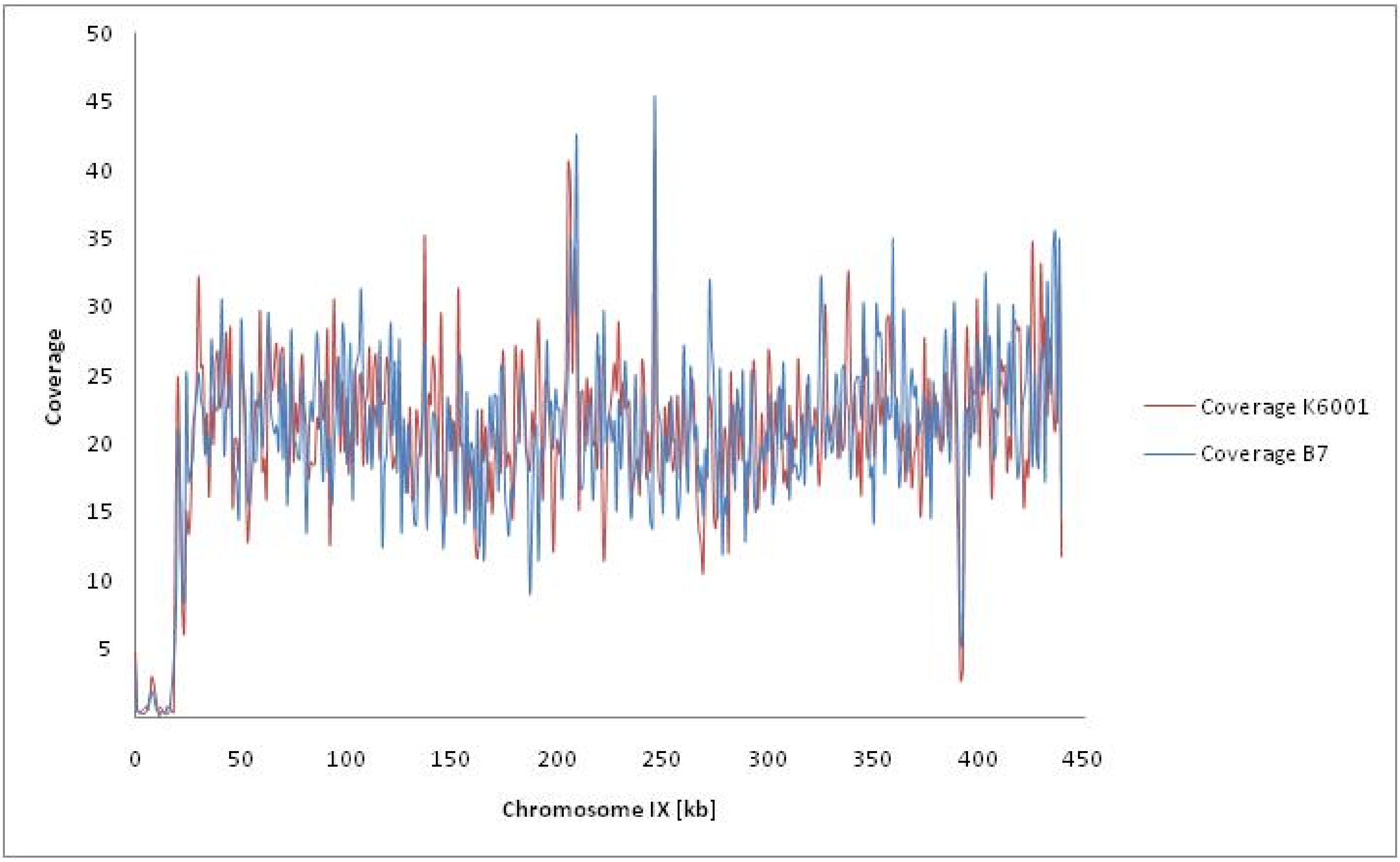
50
45
40
35
30
25
20
15
10
5
0
50
100
150
200
250
300
350
400
450
Coverage
Chromosome IX [kb]
Coverage K6001
Coverage B7

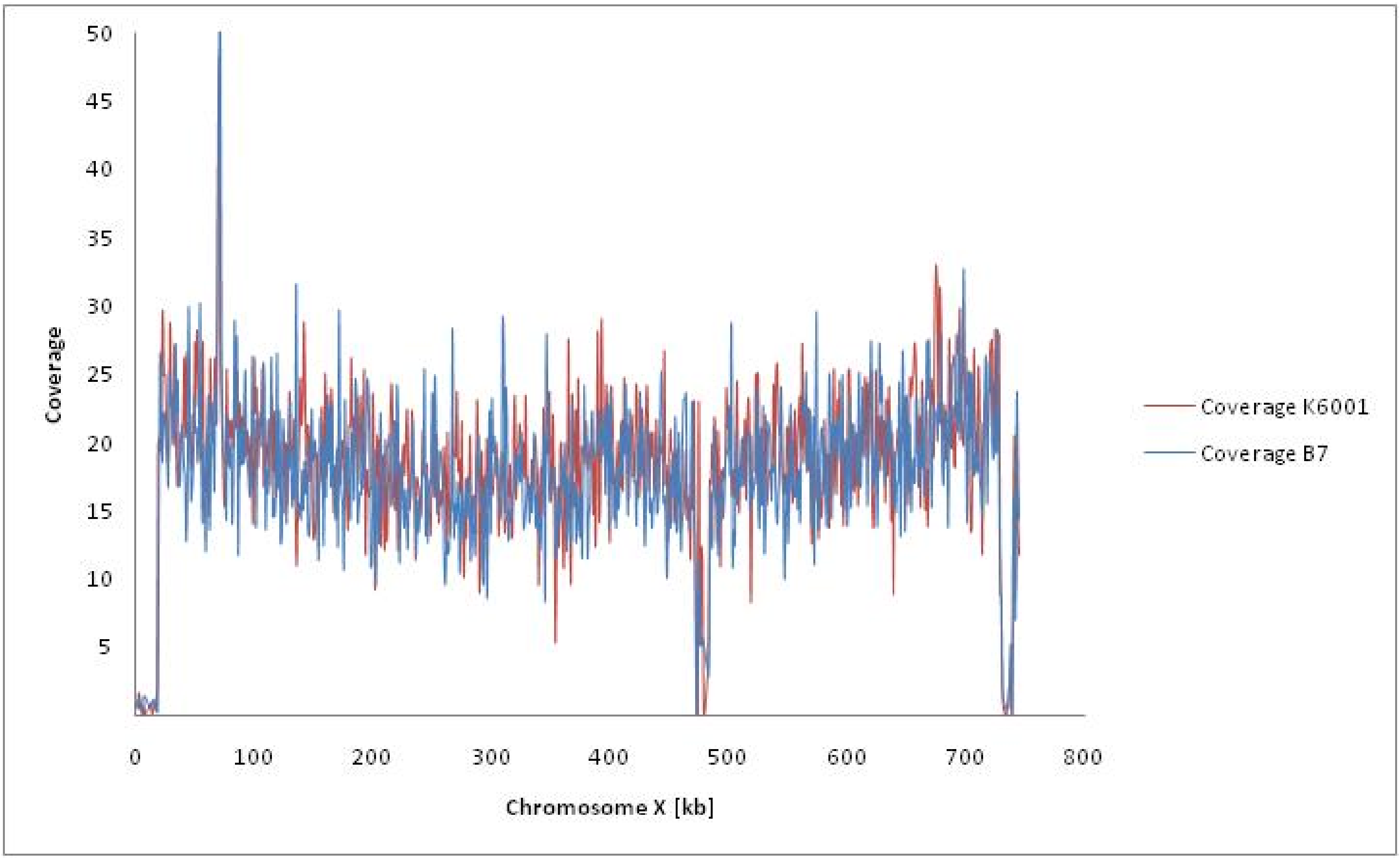
50
45
40
35
30
25
20
15
10
5
0
100
200
300
400
500
600
700
800
Coverage
Chromosome X [kb]
Coverage K6001
Coverage B7

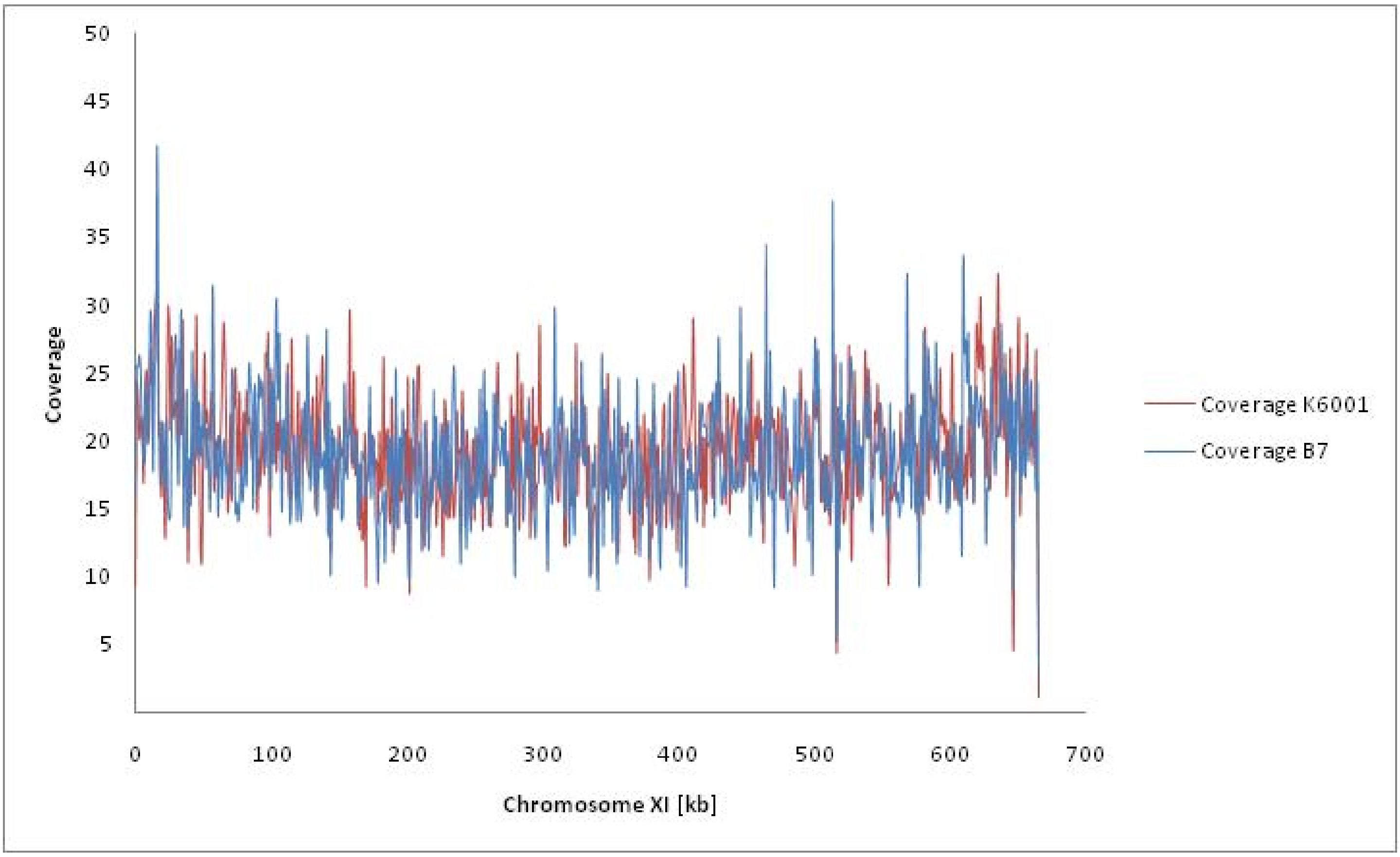
Coverage
50
45
40
35
30
25
20
15
10
5
0
100
200
300
400
500
600
700
Chromosome XI [kb]
Coverage K6001
Coverage B7

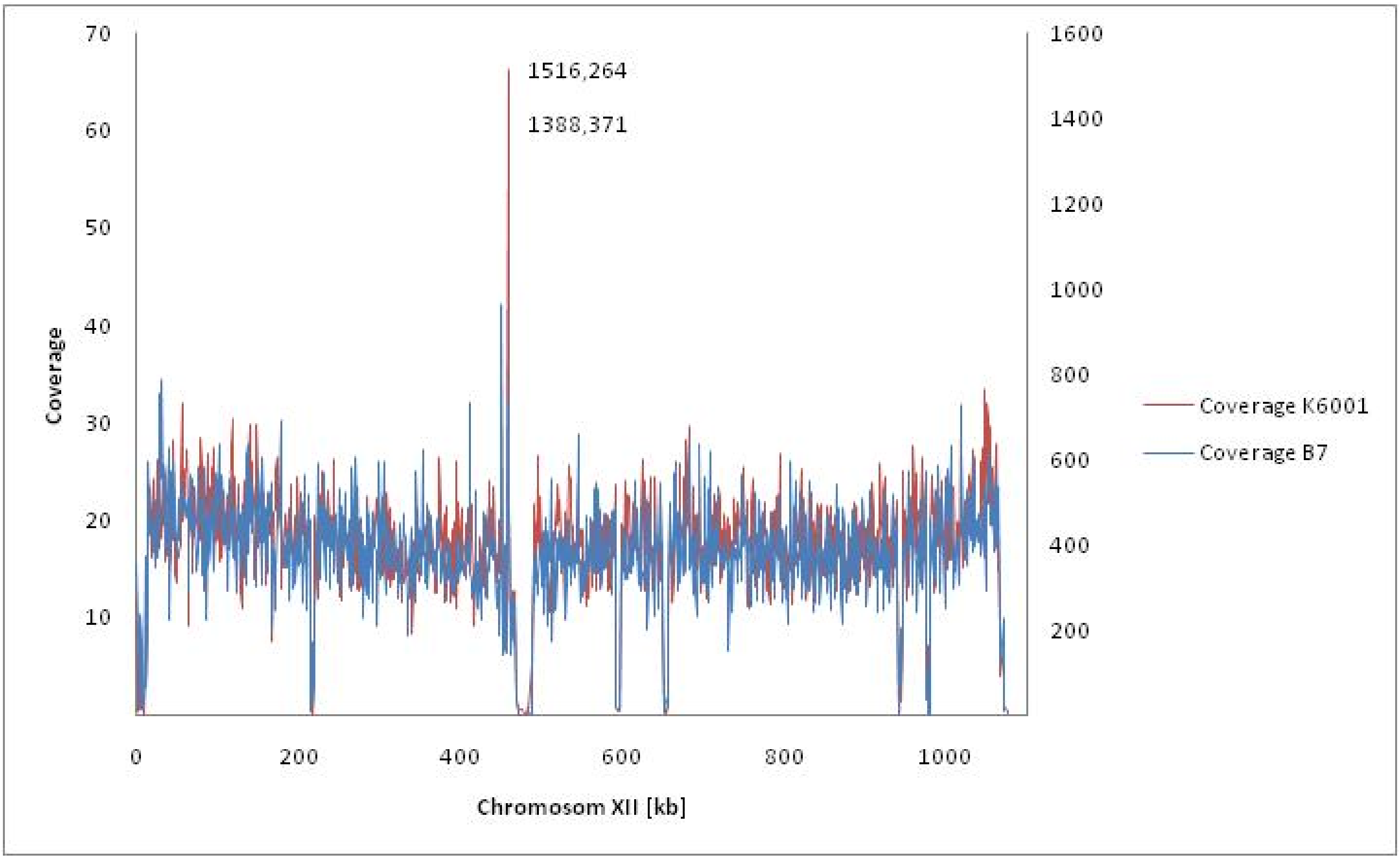
Coverage
70
60
50
40
30
20
10
1600
1400
1200
1000
800
600
400
200
1516,264
1388,371
0
200
400
600
800
1000
Chromosom XII [kb]
Coverage K6001
Coverage B7

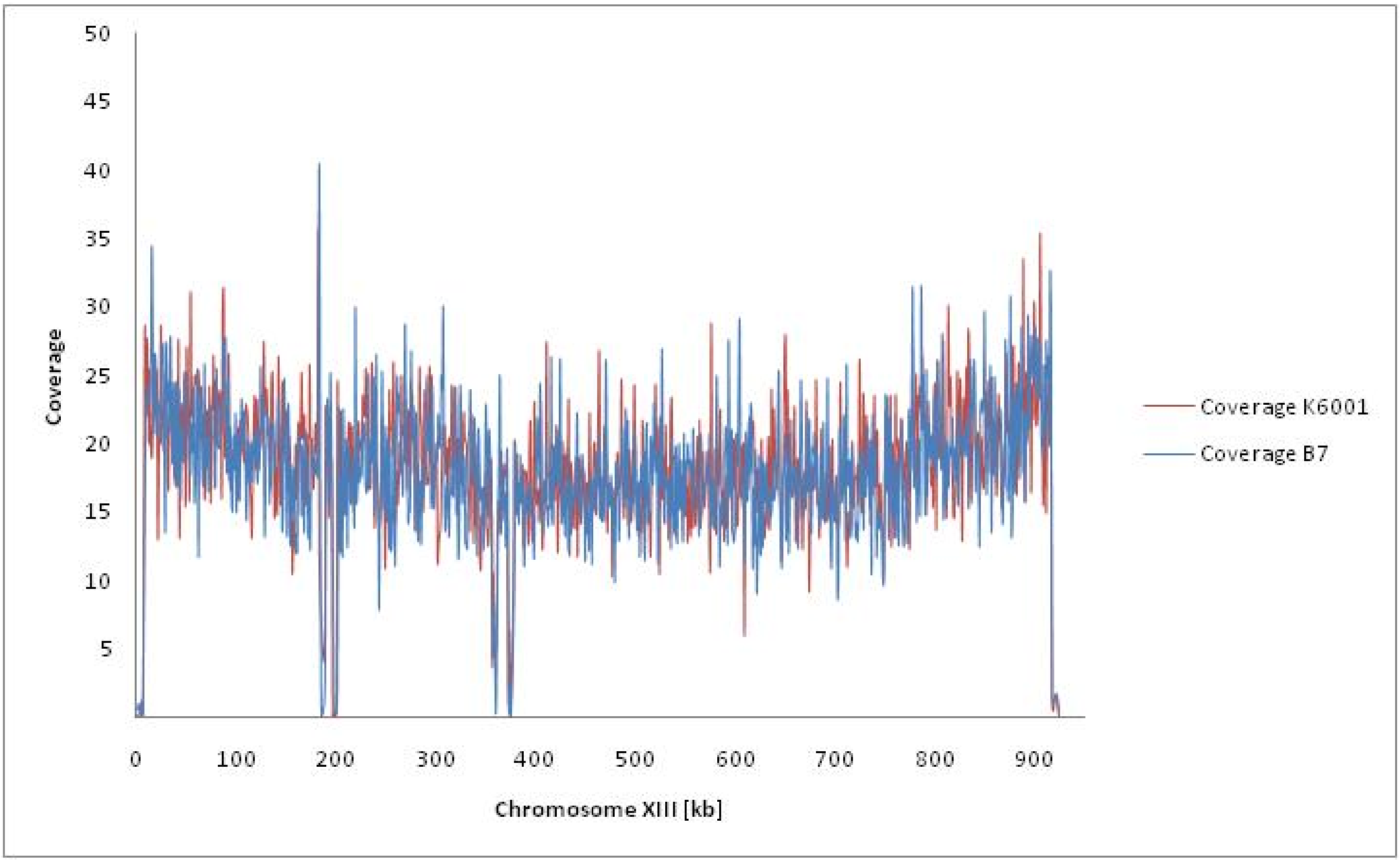

50
45
40
35
30
25
20
15
10
5
Coverage
0
100
200
300
400
500
600
700
800
900
Chromosome XIII [kb]
Coverage K6001
Coverage B7


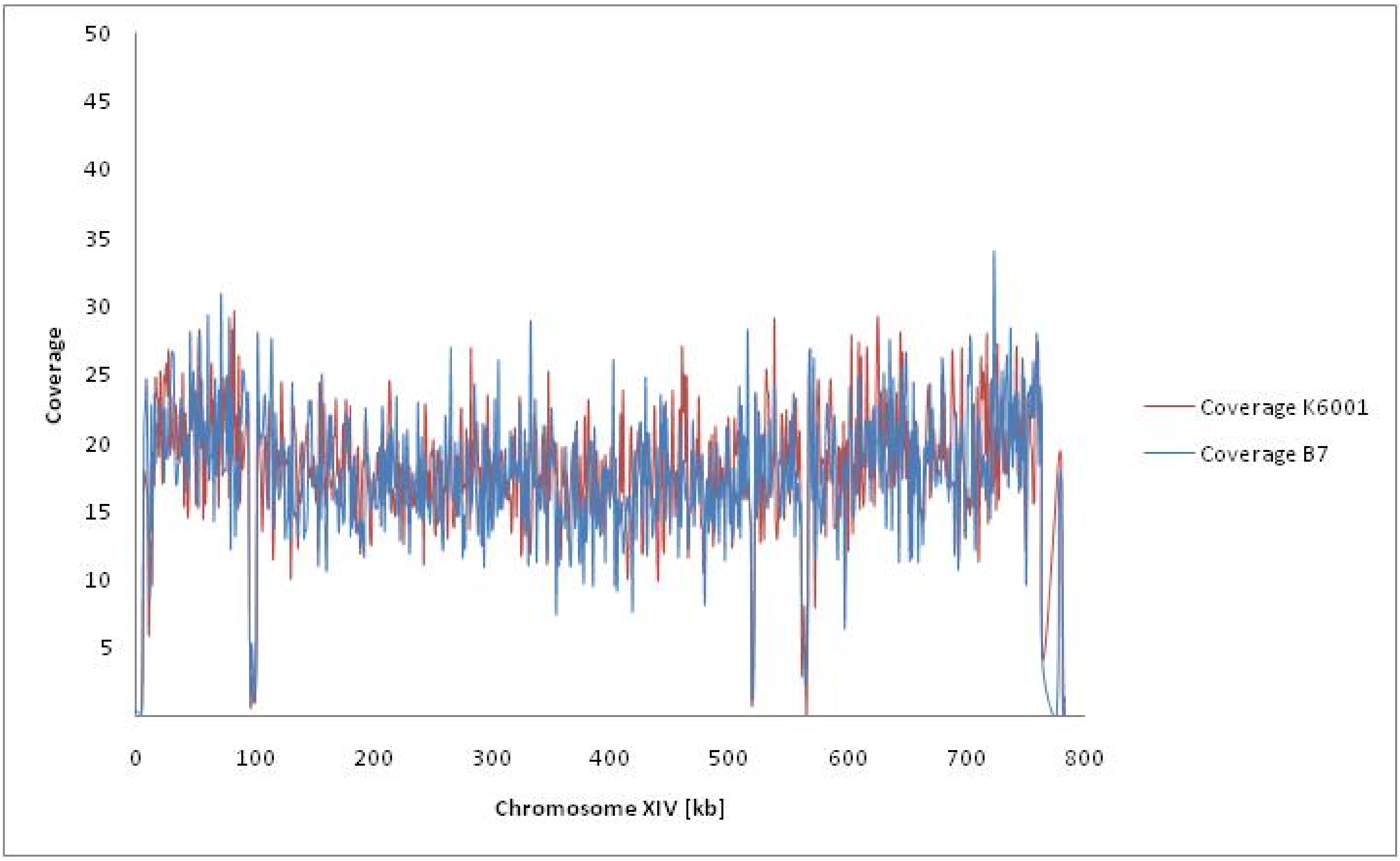

50
45
40
35
30
25
20
15
10
5
Coverage
0
100
200
300
400
500
600
700
800
Chromosome XIV [kb]
Coverage K6001
Coverage B7

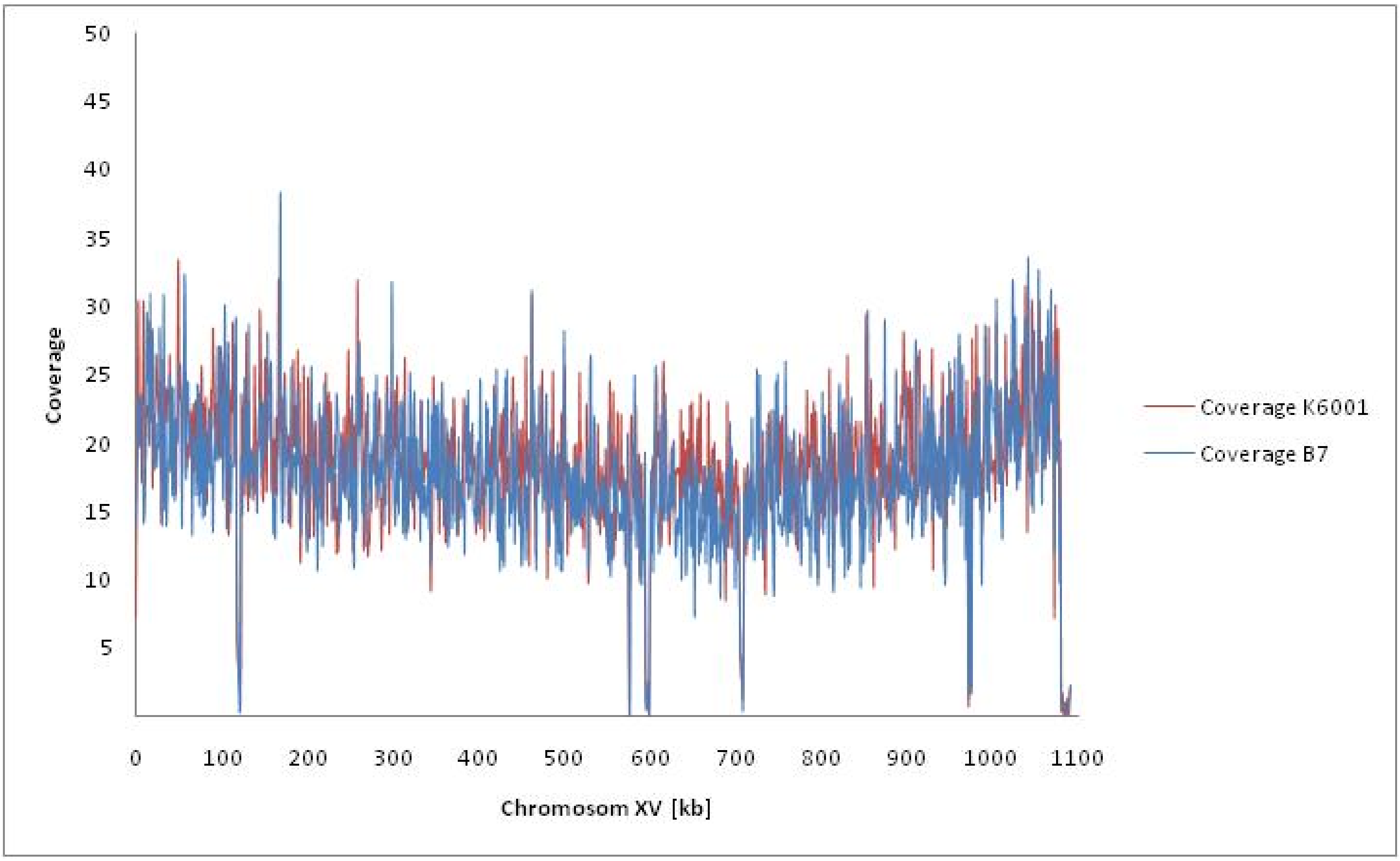
Coverage
Chromosom XV [kb]
Coverage K6001
Coverage B7
0
5
10
15
20
25
30
35
40
45
50
100
200
300
400
500
600
700
800
900
1000
1100

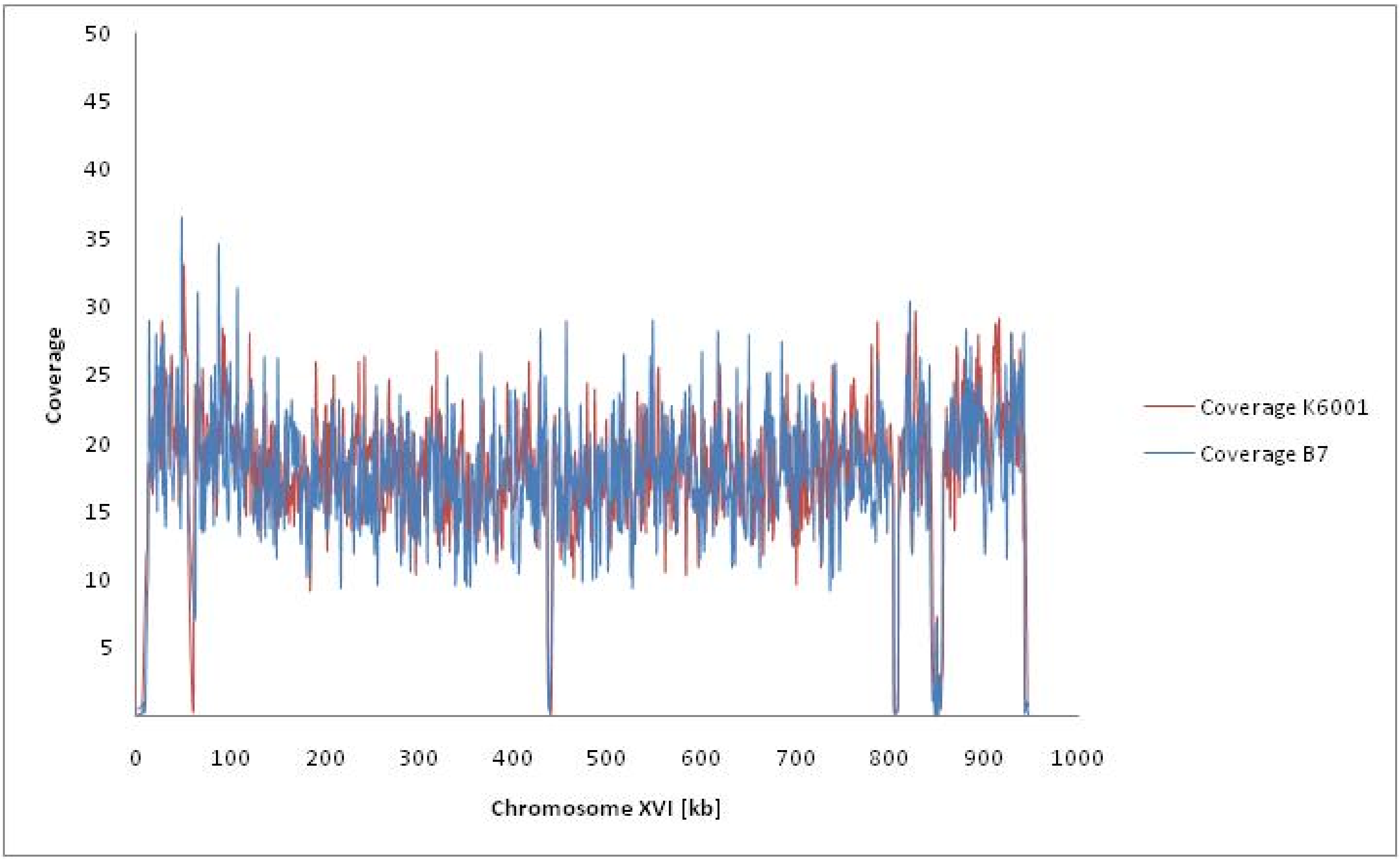
Coverage
Chromosome XVI [kb]
Coverage K6001
Coverage B7
0
5
10
15
20
25
30
35
40
45
50
100
200
300
400
500
600
700
800
900
1000
