# Supplementary material for: A new dominant peroxiredoxin allele identified by whole-genome re-sequencing of random mutagenized yeast causes oxidant-resistance and premature aging
Source: Aging (Albany NY). 2010 Aug 13;2(8):475–86. doi: 10.18632/aging.100187 (PMC2954039; doi:10.18632/aging.100187)

Supplementay Figure S2

Chromosomal distribution of *(a)* total and *(b)* amino acid exchanging SNVs that distingluish the K6001 and S288c genomes

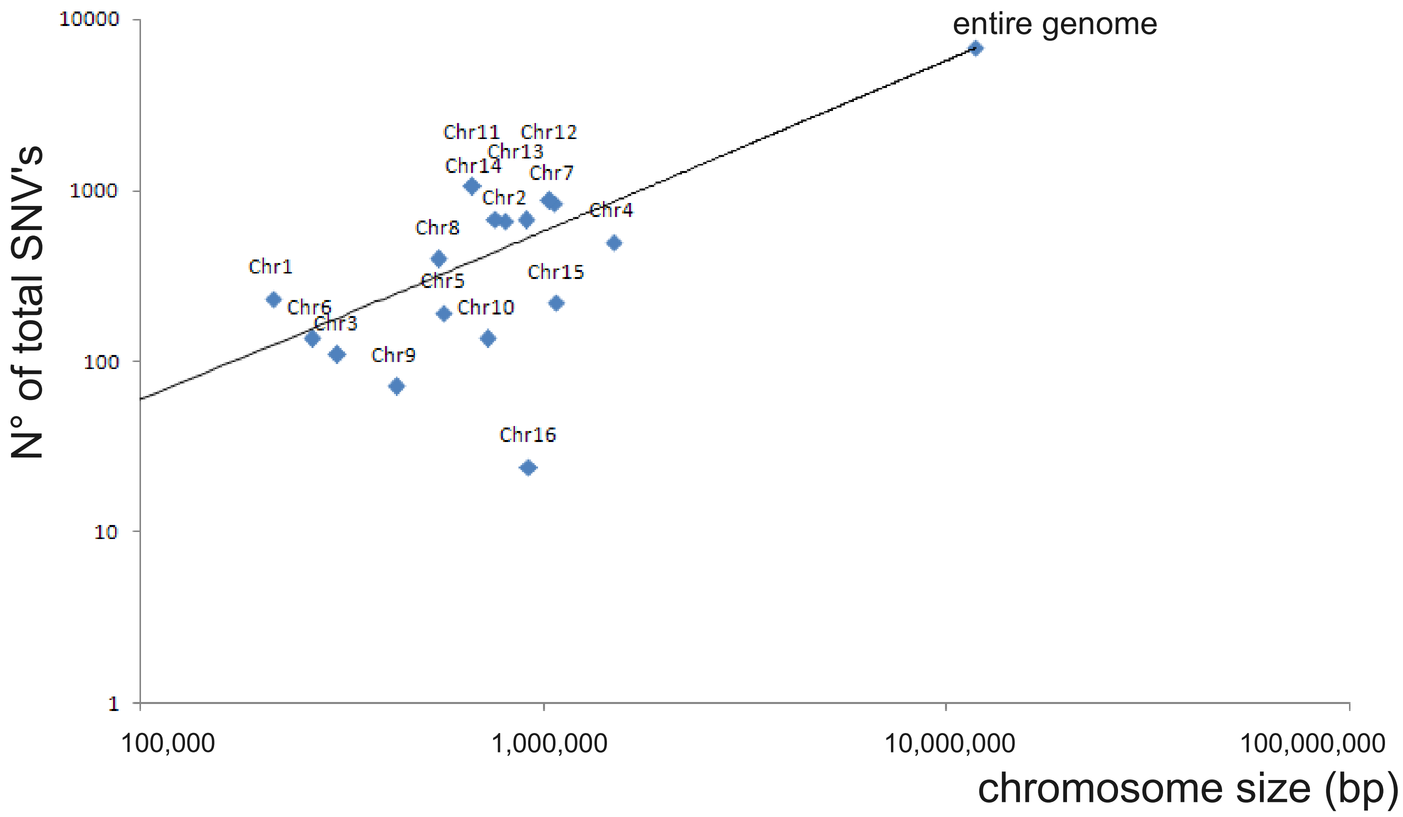

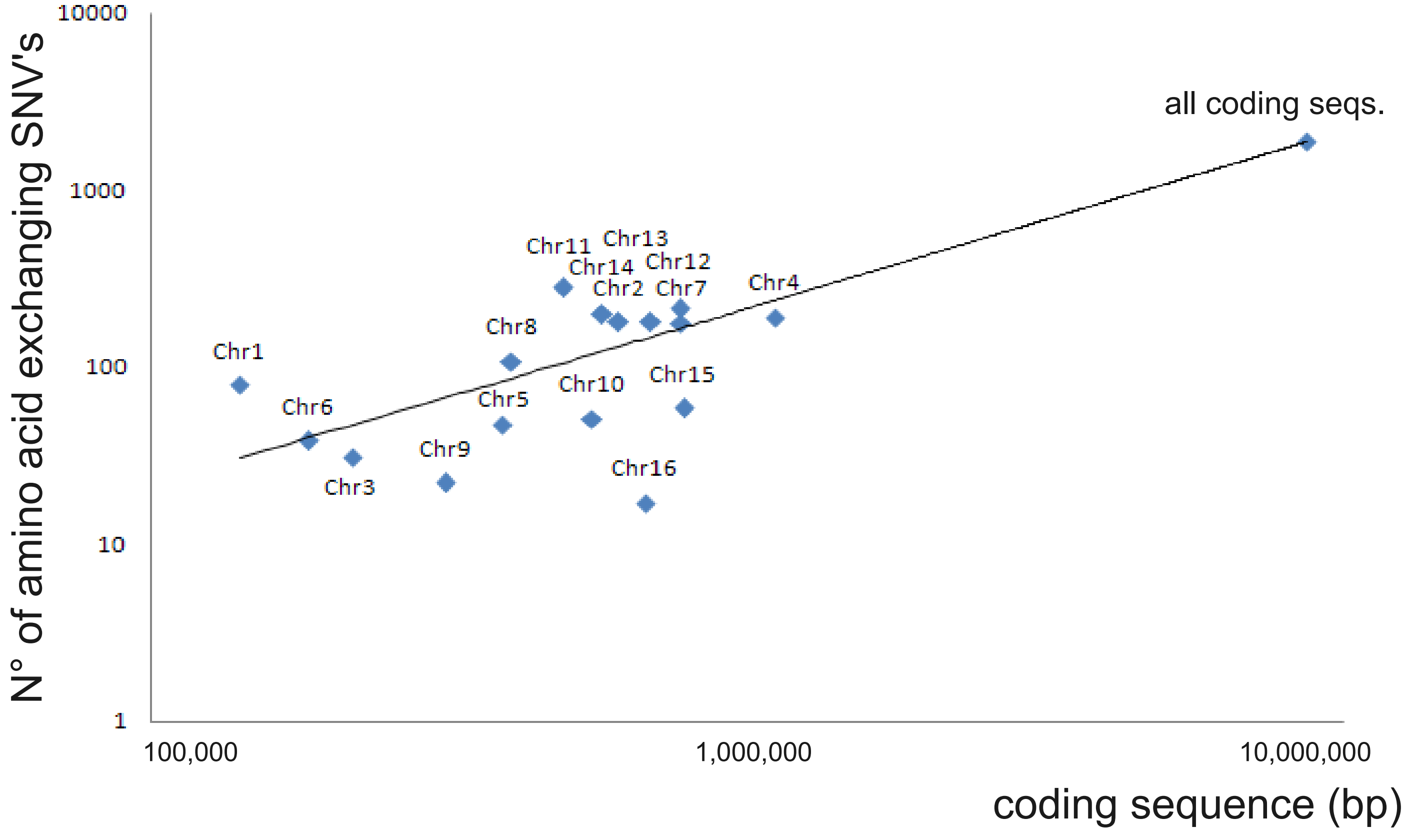

Supplement: Supplementary Figure 2 [file aging-02-475-s002.pdf]
